# Synthesis of Dense and Chiral Dendritic Polyols Using Glyconanosynthon Scaffolds

**DOI:** 10.3390/molecules21040448

**Published:** 2016-04-04

**Authors:** Tze Chieh Shiao, Rabindra Rej, Mariécka Rose, Giovanni M. Pavan, René Roy

**Affiliations:** 1 Pharmaqam and Nanoqam, Department of Chemistry, University of Québec a Montréal, P.O. Box 8888, Succ. Centre-Ville, Montréal, QC H3C 3P8, Canada; chichi_shiao@hotmail.com (T.C.S.); rabi.rej@hotmail.com (R.R.); mariecka.rose@hotmail.fr (M.R.); 2 Department of Innovative Technologies, University of Applied Sciences and Arts of Southern Switzerland, Galleria 2, Manno CH-6928, Switzerland; giovanni.pavan@supsi.ch

**Keywords:** dendrimer, glycodendrimer, carbohydrate, click chemistry, CuAAAC, thiol-yne, thiol-ene

## Abstract

Most classical dendrimers are frequently built-up from identical repeating units of low valency (usually AB_2_ monomers). This strategy necessitates several generations to achieve a large number of surface functionalities. In addition, these typical monomers are achiral. We propose herein the use of sugar derivatives consisting of several and varied functionalities with their own individual intrinsic chirality as both scaffolds/core as well as repeating units. This approach allows the construction of chiral, dense dendrimers with a large number of surface groups at low dendrimer generations. Perpropargylated β-d-glucopyranoside, serving as an A_5_ core, together with various derivatives, such as 2-azidoethyl tetra-*O*-allyl-β-d-glucopyranoside, serving as an AB_4_ repeating moiety, were utilized to construct chiral dendrimers using “click chemistry” (CuAAC reaction). These were further modified by thiol-ene and thiol-yne click reactions with alcohols to provide dendritic polyols. Molecular dynamic simulation supported the assumption that the resulting polyols have a dense structure.

## 1. Introduction

Dendrimers are hyperbranched, globular shaped, well-defined macromolecules possessing a wide range of applications in nanomedicine, novel dendrimer space concept in medicinal chemistry, as multivalent carriers for drug delivery, material sciences, catalysis, and gene therapy [1,2,3,4,5,6,7,8]. During the last three decades, there has been tremendous growth in this field motivating chemists and material scientists to conceive new ways to synthesize these molecules more efficiently, rapidly, and in a cost effective way. Most of the common synthetic methods start with low-valent scaffolds with reactive functionalities followed by the build-up of higher generation architectures using repetitive building blocks. The syntheses are accomplished either divergently or convergently. Unfortunately, the introductions of a large number of surface groups are usually achieved at the expense of high generation. To counterbalance this situation, only a handful of examples have been described to generate dendrimers with a high number of end groups at low generation, and the use of sugars (coined “glyconanosynthons”) has been recently reviewed to achieve this goal [5]. Hence, the use of carbohydrates as either core or multivalent, high valency branching building blocks is clearly immerging as an alternative approach to achieve higher numbers of surface groups in a fewer steps. This approach [9,10,11] has not been systematically investigated, particularly in regard to their inherent diversified stereochemical identities, functional group diversities and the chemical orthogonalities these molecules can offer [5].

In addition, an interesting and versatile ‘‘onion peel’’ strategy [12] has been recently added to the arsenal of sophisticated methods towards dendrimer synthesis. In this case, each layer of the dendritic macromolecule is constructed using different building blocks, rather than the same molecules, thus providing much greater flexibility in the synthetic schemes and leading to novel dendritic architectures and properties. Most importantly, an accelerated growth that necessitates less generation to arrive at high surface densities can be consequently achieved [13].

Herein, we report a facile and efficient route for dendrimer synthesis that can introduce a dense, chiral, and a large number of varied surface groups at low generation. To this end, a perpropargylated carbohydrate derivative, propargyl tetra-*O*-propargyl-β-d-glucopyranoside (**5**), constituting a dense all equatorial A_5_ core molecule, was used as a starting point. For the next layer generation, orthogonal building blocks, representing AB_4_ layers, were readily prepared using classical carbohydrate chemistry. Toward this goal, suitably functionalized 2-azidoethyl β-D-glucopyranoside derivatives were synthesized. These AB_4_ building blocks were linked to the core **5** employing highly efficient, atom economical copper-catalyzed alkyne-azide cycloaddition (“click” reaction, CuAAc). Lastly, thiol-ene and thiol-yne “click” reactions were next utilized to introduce the surface groups constituted herein of polyols.

## 2. Results and Discussion

### 2.1. Dendrimer Synthesis

**Scheme 1:**
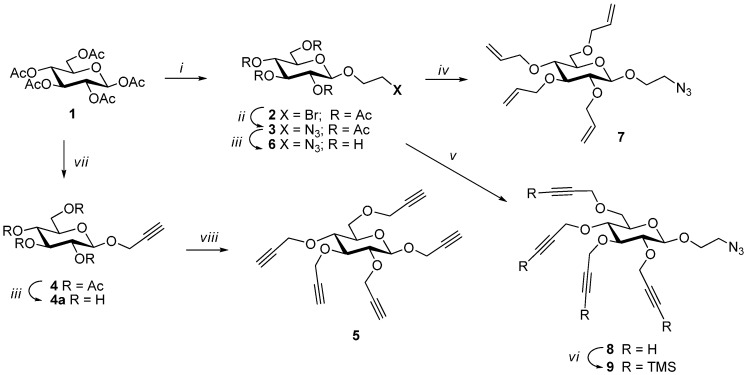
Syntheses of the glyconanosynthons. *Reagents and conditions*: (i) 2-bromoethanol, BF_3_·OEt_2_, DCM, rt, 2.5 h, 84%; (ii) NaN_3_, DMF, 85 °C, 2 h, 94%; (iii) NaOMe, MeOH, rt, 2.5 h; (iv) DMF, 60% NaH, rt, 0.5 h; allyl bromide, 0 °C, 1 h, then at rt, 3 h, 94%; (v) DMF, 60% NaH, rt, 15 min; 0 °C, propargyl bromide, 2 h, 87%; (vi) DBU, DCM, AgCl, TMSCl, 45 °C, overnight, 83%; (vii) propargyl alcohol, BF_3_·etherate, DCM, rt, 2 h, 92%; (viii) DMF, 60% NaH, 0–5 °C, 0.5 h, then propargyl bromide, 1 h at 0–5 °C, 95%.

Dendrimers were constructed in a divergent manner using propargyl 2,3,4,6-tetra-*O*-propargyl-β-d-glucopyranoside (**5**) as a pentavalent core (**A_5_**), which was synthesized from penta-*O*-acetyl-β-d-glucopyranose (**1**) (Scheme 1). Penta-*O*-acetyl-β-d-glucopyranose was prepared on a large scale by a procedure developed in our laboratory [14]. This was treated with propargyl alcohol in the presence of BF_3_·etherate in DCM yielding propargyl 2,3,4,6-tetra-*O*-acetyl-β-d-glucopyranoside [15] (**4**), which was de-*O*-acetylated under Zemplén conditions using NaOMe in MeOH. The free sugar thus obtained was treated with NaH in DMF to form the corresponding alkoxide which was perpropargylated using propargyl bromide at 0 °C to produce propargyl tetra-*O*-propargyl-β-d-glucopyranoside (**5**) at an excellent yield [16]. Other orthogonal building blocks needed for the dendrimer construction were synthesized from the same precursor. Penta-*O*-acetyl-β-d-glucopyranose was treated with 2-bromoethanol in presence of BF_3_.etherate in DCM at room temperature, which produced 2-bromoethyl 2,3,4,6-tetra-*O*-acetyl-β-d-glucopyranoside (**2**) at a good yield [17]. The bromo derivative thus obtained was converted to azido-derivative (**3**) [18] (AB_4_) by heating with sodium azide in DMF at 85 °C at an almost quantitative yield, which was used to construct the next level of the dendrimer (**G_1_**). A diagnostic peak of the azide was observed in the IR spectrum at 2104 cm^−1^. Two other building blocks were synthesized from 2-azidoethyl 2,3,4,6-tetra-*O*-acetyl-β-d-glucopyranoside (**3**) by de-*O*-acetylation under Zemplén conditions using NaOMe in MeOH. The unprotected sugar (**6**) [19] thus obtained was treated with sodium hydride in DMF to form the corresponding alkoxide, which was treated with allyl bromide to obtain 2-azidoethyl 2,3,4,6-tetra-*O*-allyl-β-d-glucopyranoside (**7**) [20] and with propargyl bromide to produce 2-azidoethyl 2,3,4,6-tetra-*O*-propargyl-β-d-glucopyranoside (**8**) at excellent yields. The propargyl functionalities of the tetra-propargylated derivative **8** were protected with trimethyl silyl group [21,22] using silver chloride, chlorotrimethylsilane, and 1,8-diazabicyclo[5.4.0]undec-7-ene DBU in DCM. TMS-derivative **9** thus obtained was used as an orthogonal building block to construct **G_1_** dendrimer generation with access to propargyl-groups for further elaboration.

**Scheme 2:**
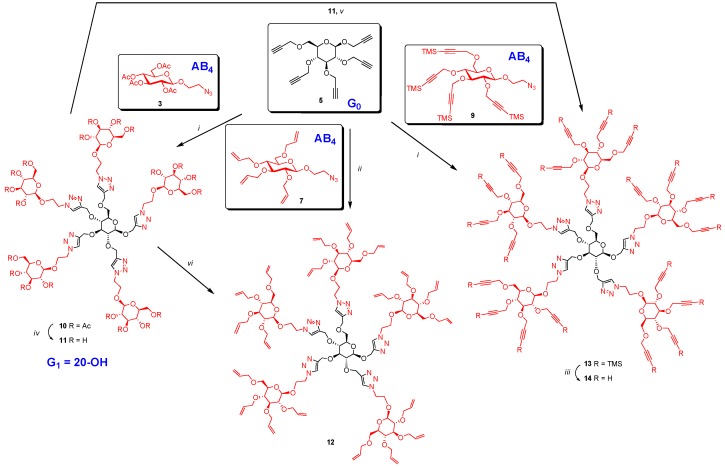
Synthesis of glycodendrimer core. *Reagents and conditions*: (i) CuI·P(OEt)_3_, toluene, 70 °C, 20 h; 85% for **10**, 90% for **13** using Hunig’s base; (ii) CuI·P(OEt)_3_, toluene, microwave, 70 °C, 4 h, 80%; (iii) TBAF, THF, acetic acid, rt, 5 h, 85%; (iv) NaOMe, MeOH-water mixture (10:1), rt, 18 h, 98%; (v) DMF, propargyl bromide, 0 °C, NaH 60% in oil added in four portions every hour, then at rt, 18 h, 70%; (vi) DMF, NaH 60% in oil, 0 °C, 1 h, then at rt, 2 h; cooled to 0 °C, allyl bromide, then at rt, 18 h, 77%.

Propargyl 2,3,4,6-tetra-*O*-propargyl-β-d-glucopyranoside (**5**) was treated with excess 2-azidoethyl 2,3,4,6-tetra-*O*-acetyl-β-d-glucopyranoside (**3**) (Scheme 2) using copper mediated CuAAC, reaction. All propargyl groups of **5** reacted with the azido-functionalities of **3** forming five distinct triazole rings. Indeed, NMR spectrum of the resulting product **10** showed four singlets at δ 8.04 (1H), 7.93 (1H) 7.88 (2H), 7.71 (1H) and absence of any residual propargyl signal. Similarly, excess of 2-azido ethyl 2,3,4,6-tetra-*O*-allyl-β-d-glucopyranoside (**7**) was treated with propargyl 2,3,4,6-tetra-*O*-propargyl-β-d-glucopyranoside (**5**) under ‘‘Click’’ condition using CuI·P(OEt)_3_ [16] in dry toluene at 70 °C under microwave yielding perallylated product **12**. The presence of five signals at δ 8.08, 8.03, 7.98, 7.93 and 7.80 ppm due to five triazole rings and absence of signal at 2.41–2.51 ppm due to propargyl groups in the NMR spectrum indicated complete conversion. Dendrimer (**10**) was de-*O*-acetylated under Zemplén condition using NaOMe in MeOH-water mixture (9:1) yielding perhydroxylated compound (**11**). This served as a common intermediate for the synthesis of both perallylated (**12**) and perpropargylated **14** derivatives. Perallylation [23] of (**11**) was accomplished by forming the anion from **11** using NaH in DMF first, then by reacting with excess allyl bromide at room temperature for 18 h. Perallylated **12** was obtained in good yield (77%) and was identical in TLC and NMR with the product obtained from the ‘‘Click’’ reaction involving **5** and **7**. Moreover, it also showed a perfect match in its ESI-HRMS spectrum.

**Figure 1:**
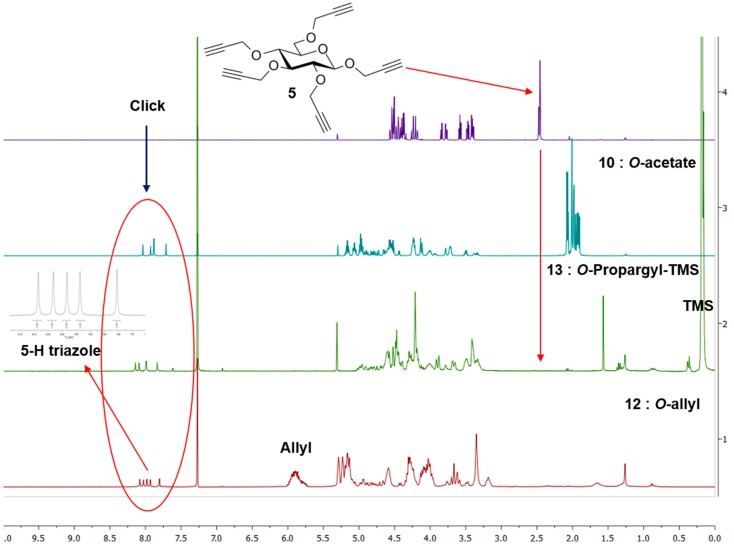
NMR spectra of key precursor **5** and **G_1_** dendrimers **10**, **12** and **13**. Insert: Expanded triazole signals of **12**.

Excess of TMS-protected propargyl derivative **9** was treated with perpropargylated glucopyranoside **5** under “Click” condition using soluble copper reagent (CuI·P(OEt)_3_) in the presence of Hunig’s base [22] at 70 °C for 20 h affording per-TMS-propargylated dendrimer **13** in 90% yield. The use of Hunig’s base was necessary to avoid loss of TMS groups during the reaction and to improve the yield. TMS-protected propargyl ethers were removed by treatment with tetrabutylammonium fluoride (TBAF) [22] buffered with acetic acid in THF, yielding perpropargylated dendrimer (**14**) in 85% yield. The same perpropargylated product was obtained by direct propargylation [24] of the anion derived from **11** by treating with NaH in DMF in presence of excess propargyl bromide in acceptable yield (70%). Both the products were identical by TLC and by NMR spectroscopy (Figure 1) and showed once again a perfect ESI-HRMS spectrum.

**Scheme 3:**
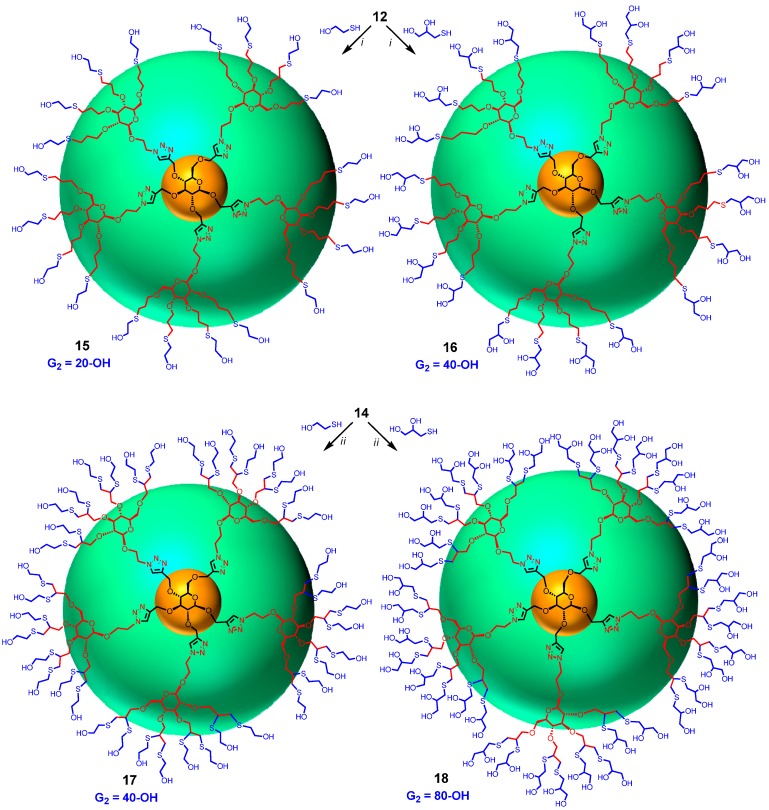
Syntheses of dendritic polyols. *Reagents and conditions*: (i) AIBN, dioxane, 100°C, **15**, 47%, **16**, 60%; (ii) UV 365 nm, DMPA, 4 h and 6 h; **17**, 56% and **18**, 71%.

**Scheme 4:**
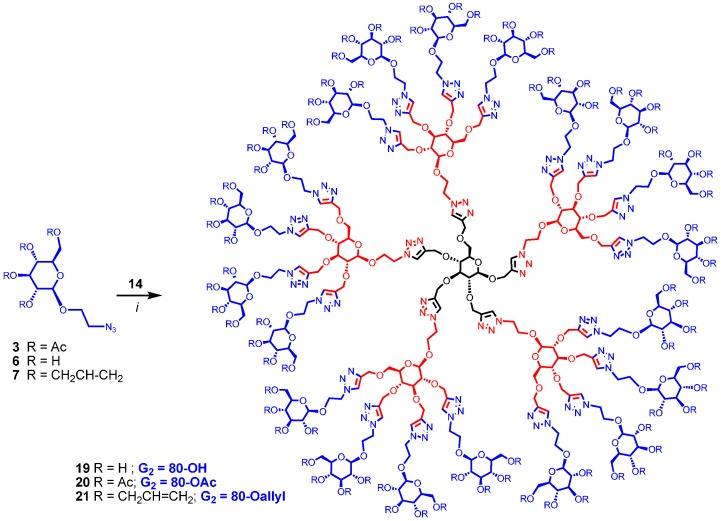
Synthesis of G2 dendrimers. *Reagents and conditions*: (i) CuI·P(OEt)_3_, DMF, 70 °C, 20 h; 53% for **19**; 67% for **20**; 57% for **21**.

Finally, dendrimer **12** obtained above was progressed to the **G_2_** generation by thiol-ene click reaction using 2-mercaptoethanol and thioglycerol by heating with catalytic amount of azobisisobutyronitrile (AIBN) in dioxane [25] at 100 °C yielding dendritic polyols **15** and **16** having 20 and 40 hydroxyl groups, respectively (Scheme 3). Completion of the reaction was monitored by the clear and complete disappearance of the signal at 5.72–5.98 δ due to allyl groups. Similarly, intermediate **14** was converted into dendrimers **17** and **18** containing 40 and 80 hydroxyl surface-groups by irradiating under UV (365 nm) with 2-mercaptoethanol and thioglycerol in the presence of dimethoxyphenyl acetophenone (DMPA). The reactions were monitored by the disappearance of all the propargyl signals at δ 2.40–2.60 ppm. Thus, by introducing sugar at the core (**G_0_**) as well as at the **G_1_** level, large number of surface groups was incorporated into the dendrimer in just two steps from the monomeric precursors. Sugar units were also introduced at the **G_2_** level of the dendrimer by treating **14**, having 20 propargyl groups, with 2-azidoethyl β-d-glucopyranoside (**6**) using CuAAC click reaction with soluble CuI·P(OEt)_3_ as catalyst in DMF at 65–70 °C for 20 h yielding dendrimer **19** with 80 hydroxyl end-groups (Scheme 4). Similarly, compound **20**, with 80 acetyl surface-groups, can be easily prepared from the same precursor (**14**) and 2-azidoethyl 2,3,4,6-tetra-*O*-acetyl-β-d-glucopyranoside (**3**) in an efficient way using click reaction. Lastly, 80 allyl end-groups were introduced into the dendrimer by treating **14** with 2-azidoethyl 2,3,4,6-tetra-*O*-allyl-β-d-glucopyranoside (**7**) under the click reaction to give **21** in good yield (57%). Reactions were monitored by the complete disappearance of all propargyl signals of **14** at δ 2.40–2.60 ppm.

All monomeric building blocks were properly characterized by NMR (^1^H, ^13^C and COSY), IR and mass spectrometry. Dendrimers **10**, **11**, **12** and **14** showed correct mass corresponding to their molecular formula in HRMS and exhibited good ^1^H-NMR and ^13^C-NMR in agreement with the assigned structures. All dendrimers **15**, **16**, **17**, **18**, **19**, **20**, and **21** showed molecular ions in MALDI-TOF, though in some cases M^+^ signals were broad signals, likely due to the facile loss of water molecules or the presence of varied cations (Na^+^, K^+^, NH_4_^+^) in the polyols. Dendrimers **20** and **21** exhibited good GPC curves (single peaks) indicating their mono-dispersity (see Supplementary Materials).

### 2.2. Molecular Dynamics Simulations

**Figure 2:**
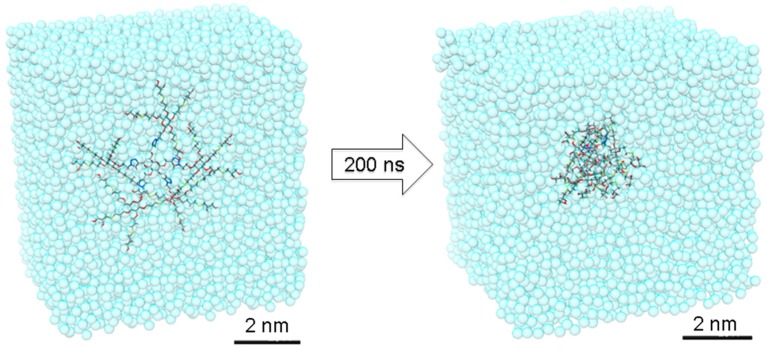
The MD simulation of **16**, starting from an initially fully extended conformation. Dendrimer **16** rapidly rearranges into a 2 nm stable globular shape in water.

**Figure 3:**
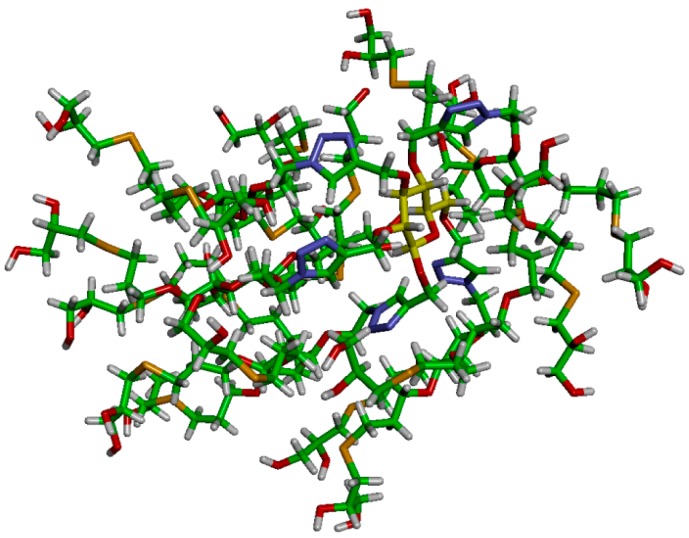
Stick representation of dendrimer **16** after equilibration by MD simulation (water molecules not shown).

In order to gain additional insight into these interesting dense nanostructures, we created an atomistic model for dendrimer **16** and have simulated it in a box filled of explicit water molecules by means of all-atom molecular dynamics (MD) simulation. The initially extended conformation of **16** has been equilibrated during 200 ns of MD simulation. During this time, the dendrimer folded assuming a quasi-globular shape (Figure 2 and Figure 3). The radius of gyration of **16** was found to be R_g_ = 9.8 ± 0.5 Å, while the strong folding was found to be stable during the MD simulation (see Supplementary Materials).

**Figure 4:**
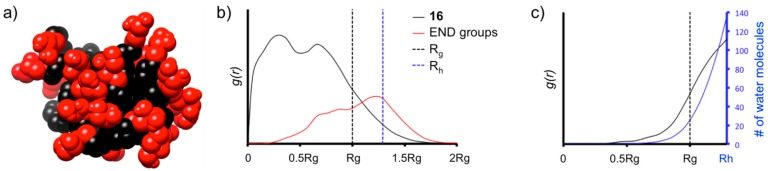
MD characterization of dendrimer **16** in water. (**a**) Equilibrated configuration of **16**—black: scaffold, red: OH-surface groups; (**b**) radial distribution functions g(r) of the atoms of **16** (black) and of the surface OH-groups (red) as a function of the distance from the dendrimer’s center of mass. The hydrodynamic radius of **16** is calculated according to the model of a rigid sphere as: R_h_ ≈ 1.29 R_g_ [28]; (**c**) radial distribution functions, *g(r)*, of the water molecules (black), and number of water molecules (blue) as a function of the distance from **16**’s center of mass.

From the equilibrated phase MD trajectories, we obtained the radial distribution functions (*g(r)*) of the atoms of **16**, of the surface groups, and of the water molecules calculated using the dendrimer’s center of mass (Figure 4). The *g(r)* curves are indicative of the probability density for finding certain groups of the dendrimer in space and are useful to understand the level of density in the various regions of the dendrimer structure [26], of hydration and water penetration [27,28] into the scaffold and also of the displacement of the surface groups at the dendrimer’s surface both in terms of surface groups crowding and directionality [29,30].

It is interesting to note that the high level of branching in the scaffold of **16** produces high-density in the dendrimer’s interior and reduced surface group backfolding. Seen in Figure 4b, the *g(r)* of the surface OH-groups of the dendrimer (red) are most probably found at greater distance from the dendrimer’s center than all other atoms of **16**. This indicates that the surface groups well surround the dendrimer scaffold. This picture of high interior density is well consistent with the *g(r)* of the water molecules (Figure 4c), demonstrating that water penetration is present only close to the surface, identified by R_g_ (no water penetration for R < 0.5 R_g_).

Table S1 reports other interesting structural data for **16**. In particular, comparison between dendrimer’s R_g_ and solvent accessible surface area (SASA) provides an interesting cue on the level of porosity of this dendrimer. Namely, SASA is the surface in contact with the solvent. From this parameter, it is possible to obtain R_SASA_, which is the radius of a sphere having surface equivalent with that of the dendrimer in contact with the solution as: R_SASA_ = sqrt(SASA/4π). R_SASA_ is larger than R_g_, because the dendrimer is not a rigid sphere but a porous soft macromolecule with a non-perfect surface. Comparison between the volume of the voids in the dendrimer and the full one leads to a quantification of **16**’s porosity (0.78, see Table S1), which is slightly higher than that of Polyamidoamine (PAMAM) G2 dendrimer (0.75), having SASA ≈ 3.5 × 10^3^ Å^2^ and R_g_ = 10.4 Å^3^. Compared to G2 PAMAM, the data from the MD simulation suggest that **16** is more heavy and dense in the interior even if having a larger surface. This is consistent with the fact that **16** is slightly smaller than a G2 PAMAM (slightly smaller R_g_) while the MW is higher (4581 Da for **16** *vs.* 3256 Da for G2 PAMAM) and it has a larger number of surface groups: 20 *vs.* 16.

## 3. Materials and Methods

All reactions in organic medium were performed in standard oven dried glassware under an inert atmosphere of nitrogen using freshly distilled solvents stored over molecular sieves. Solvents were deoxygenated when necessary by bubbling nitrogen through the solution. All reagents were used as supplied without prior purification and obtained from Sigma-Aldrich Chemical Co. (Toronto, ON, Canada) Reactions were monitored by analytical thin-layer chromatography (TLC) using silica gel 60 F254 precoated plates (E. Merck, Darmstadt, Germany) and compounds were visualized by 254 nm light and/or by dipping into a mixture of sulfuric acid and methanol in water or into a mixture of KMnO_4_ and K_2_CO_3_ in water followed by gentle warming with a heat-gun. Purifications were performed by flash column chromatography using silica gel from Canadian Life Science (60 Å, 40–63 µm) (Peterborough, ON, Canada) with the indicated eluent. ^1^H-NMR and ^13^C-NMR spectra were recorded at 300 and/or 600 MHz and 75 and/or 150 MHz, respectively, on a Bruker spectrometer (300 MHz and 600 MHz) (Milton, ON, Canada) and Varian spectrometer (600 MHz) (Milton, ON, Canada). All NMR spectra were measured at 25 °C in indicated deuterated solvents. Proton and carbon chemical shifts (δ) are reported in ppm and coupling constants (J) are reported in Hertz (Hz). The resonance multiplicity in the ^1^H-NMR spectra are described as “s” (singlet), “d” (doublet), “t” (triplet), and “m” (multiplet) and broad resonances are indicated by “broad”. Residual protic solvent of CDCl_3_ (^1^H, δ 7.27 ppm; ^13^C, δ 77.0 ppm (central resonance of the triplet)), D_2_O (^1^H, δ 4.80 ppm and 30.9 ppm for CH_3_ of Acetone for ^13^C spectra), MeOD (^1^H, δ3.30 ppm and ^13^C, δ 49.0 ppm). Two-dimensional homonuclear correlation ^1^H-^1^H COSY experiments were used to confirm NMR peak assignments. Fourier transform infrared (FTIR) spectra were obtained with Thermo-scientific, Nicolet model 6700 (Mississauga, ON, Canada) equipped with Alternate Total Reflection (ATR). The absorptions are given in wave numbers (cm^−1^). Accurate mass measurements (HRMS) were performed on a LC-MSD-TOF instrument from Agilent Technologies (Santa Clara, CA, USA) in positive electrospray mode. Either protonated molecular ions [M + nH]^n+^ or adducts [M + nX]^n+^ (X = Na, K, NH_4_) were used for empirical formula confirmation. Gel Permeation Chromatography (GPC) was performed using chloroform and THF as the eluent, at 40 °C with a 1 mL/min flow rate on a Viscotek VE 2001 GPCmax (SEC System) (Viscotek/Malvern Instruments Ltd, Worcestershire, UK) with Wyatt DSP/Dawn EOS (Wyatt Technology, Santa Barbara, CA, USA) and refractive index RI/LS system as detectors (Wyatt Technology, Santa Barbara, CA, USA). In addition, 2 PLGel mixed B LS (10 μm, 300 × 7.5 mm) and Light Scattering-Multiangle Laser Light Scattering (LS-MALLS) detection with performances verified with polystyrene 100 kDa and 2000 kDa were used to determine the number-average molecular weight (*M*_n_) and polydispersity index (*M*_W_/*M*_n_). Calculations were performed with Zimm Plot (model).

Syntheses of 1,2,3,4,6-penta-*O*-acetyl-β-d-glucopyranose (**1**), 2-bromoethyl 2,3,4,6-tetra-*O*-acetyl-β-d-glucopyranoside (**2**), 2-azidoethyl 2,3,4,6-tetra-*O*-acetyl-β-d-glucopyranoside (**3**), 2-azidoethyl 2,3,4,6-tetra-*O*-allyl-β-d-glucopyranoside (**7**) were performed following the literature procedures in good yields.

*Propargyl 2,3,4,6-tetra-O-propargyl-β-d-glucopyranoside* (**5**). To a solution of prop-2-ynyl-β-d-glucopyranoside (**4a**) (310 mg, 1.42 mmol) in DMF (5 mL) cooled to 0 °C was added sodium hydride (60% in oil, 530 mg, 13.2 mmol). The mixture was stirred for 30 min at this temperature. Propargyl bromide (3 mL, 20.2 mmol) was added dropwise. The mixture was stirred at 0–5 °C for 1 h. It was quenched with NH_4_Cl solution. It was extracted with ethyl acetate and washed with brine. The extract was dried over sodium sulfate and evaporated under vacuum. The crude product was chromatographed over silica gel (hexane-ethyl acetate (0–30%) as eluent) yielding **5** as light yellow oil (500 mg, 1.35 mmol, 95%). R*_f_* = 0.22 (Hexanes/EtOAc 2:1); ^1^H-NMR (600 MHz, CDCl_3_): δ (ppm) 4.59–4.32 (m, 9H, H-1 and OC*H*_2_), 4.28–4.17 (m, 2H), 3.84 (dd, 1H, ^3^*J*_6a,5_ = 1.9 Hz, ^3^*J_6a_*_,6b_ = 10.8 Hz, *H*-6a), 3.77 (dd, 1H, ^3^*J*_6b,5_ = 4.7 Hz, ^3^*J*_6b,6a_ = 10.8 Hz, *H*-6b), 3.58 (dd, 1H, ^3^*J*_3,2_ = 8.8 Hz, ^3^*J_3_*_,4_ = 9.5 Hz, *H*-3), 3.51–3.37 (m, 3H) and 2.51–2.41 (m, 5H). ^13^C-NMR (75 MHz, CDCl_3_): δ (ppm) 100.7 (*C*-1), 83.2, 80.9, 80.0, 79.9, 79.7, 79.5, 78.6, 75.9, 75.1, 74.7, 74.4, 74.3, 74.2, 74.1, 68.3, 60.2, 60.0, 59.3, 58.6, 55.9. FT-IR: ν_max_ (neat)/cm^−1^ 3289 (br, C≡CH) and 2119 (≡CH). ESI^+^-HRMS: [M + H]^+^ calcd for C_21_H_23_O_6_, 371.1467; found: 371.1480.

*2-Azidoethyl 2,3,4,6-tetra-O-propargyl-β-d-glucopyranoside* (**8**). 2-azidoethyl 2,3,4,6-tetra-*O*-acetyl-β-d-glucopyranoside (300 mg, 0.72 mmol) was dissolved in dry methanol (6 mL) and NaOMe (1.1 M) was added slowly to bring pH to 9. The mixture was stirred at room temperature for 2.5 h. Acidic resin (IR-120) was added to neutralize the base. The mixture was filtered and evaporated to obtain fully deprotected glucoside derivative. Before being used in the next step, it was placed under vacuum for 1 h. It was dissolved in DMF (3 mL) and the solution was cooled to 0 °C. Sodium hydride (60% in oil, 240 mg, 6.0 mmol) was added. The mixture was stirred at 0 °C for 15 min. Propargyl bromide (80% in toluene, 1.5 mL, 10.1 mmol) was added dropwise. The mixture was stirred at 0 °C for 1 h. Saturated NH_4_Cl solution was added to quench the reaction. It was extracted with ethyl acetate. The extract was washed with brine, dried over MgSO_4_ and evaporated. 2-Azidoethyl 2,3,4,6-tetra-*O*-propargyl-β-d-glucopyranoside (**8**) was obtained by flash chromatography over silica gel (hexane-ethyl acetate (0–30%) as eluent) as light yellow oil (250 mg, 0.62 mmol, 87%). ^1^H-NMR (300 MHz, CDCl_3_) δ (ppm) 4.65–4.50 (m, 3H), 4.50–4.37 (m, 3H), 4.34 (d, *J* = 7.8 Hz, 1H), 4.31–4.16 (m, 2H), 4.04 (ddd, *J* = 10.6, 5.4, 3.8 Hz,1H), 3.86 (dd, *J* = 10.7, 1.8 Hz, 1H), 3.79 (d, *J* = 4.6 Hz, 1H), 3.77–3.65 (m, 1H), 3.63–3.54 (m, 1H), 3.52–3.35 (m, 5H), 2.53–2.42 (m, 4H); ^13^C NMR (150 MHz, CDCl_3_) δ (ppm) 103.1, 83.3, 81.2, 80.0, 79.9, 79.8, 79.5, 76.1, 74. 7, 74.4, 74.3, 74.30, 74.2, 68.5, 68.2, 60.3, 60.0, 59.5, 58.7, 50.9. FT-IR: ν_max_ (neat)/cm^−1^ 3290s (*br*, C≡CH), 2104 (azide). ESI^+^-HRMS: [M + NH_4_]^+^ calcd for C_20_H_27_N_4_O_6_, 419.1915; found: 419.1925.

*2-Azidoethyl 2,3,4,6-tetra-O-trimethylsilylpropargyl-β-d-glucopyranoside* (**9**) A mixture of 2-azidoethyl 2,3,4,6-tetra-*O*-propargyl-β-d-glucopyranoside (**8**) (700 mg, 1.74 mmol), AgCl (300 mg, 2.1 mmol) and DBU (3.5 g, 23 mmol) in DCM (25 mL) was brought to 40 °C. Chlorotrimethyl silane (3.1 mL, 24.4 mmol) was added dropwise. It was stirred at 45 °C for 18 h. The reaction mixture was diluted with DCM/H_2_O (300 mL, 2:1 *v*/*v*) and extracted with DCM (200 mL). The extract was washed with water (50 mL) and with brine (50 mL), dried and evaporated. The crude material was passed through a column of silica gel (hexane-ethyl acetate (0–10%) as eluent) to obtain 2-azidoethyl 2,3,4,6-tetra-*O*-trimethylsilylpropargyl-β-d-glucopyranoside (**9**) as colorless oil (1.0 g, 1.45 mmol, 83%). ^1^H-NMR (300 MHz, CDCl_3_) δ (ppm) 4.63–4.28 (m, 7H), 4.22 (s, 2H), 4.03 (ddd, *J* = 10.6, 5.5, 4.0 Hz, 1H), 3.93 (d, *J* = 10.0 Hz, 1H), 3.76–3.60 (m, 2H), 3.60–3.39 (m, 5H), 3.33 (dd, *J* = 8.9, 7.9 Hz, 1H), 0.19 (s, 35H); ^13^C-NMR (150 MHz, CDCl_3_) δ (ppm) 103.1, 102.1, 102.0, 101.8, 101.5, 91.5, 91.2, 91.1, 90.9, 83.7, 81.7, 75.9, 74.5, 69.3, 68.2, 61.0, 60.5, 60.4, 59.5, 50.9, −0.1. FT-IR: ν_max_ (neat)/cm^−1^ 2176 (substituted propargyl) and 2103 (azide). ESI^+^-HRMS: [M + NH_4_]^+^ calcd for C_32_H_59_N_4_O_6_Si_4_, 707.3487; found: 707.3506.

**Synthesis of 10.** A mixture of **5** (13 mg, 0.035 mmol), 2-azidoethyl 2,3,4,6-tetra-*O*-acetyl-β-d-glucopyranoside (**3**) (97 mg, 0.233 mmol) and CuI·P(OEt)_3_ (7 mg, 0.02 mmol) in dry toluene (2 mL) was held at 70 °C with stirring for 20 h. It was cooled and diluted with ethyl acetate (100 mL). The solution was washed with EDTA (2 × 5 mL) and with a mixture of aqueous NaHCO_3_ and brine (10 mL), dried and evaporated. The crude material was passed through a column of silica gel with hexane-ethyl acetate (1:1) and 0–6% MeOH in DCM as eluents yielding **10** as colorless oil (73 mg, 0.03 mmol, 85%). ^1^H-NMR (600 MHz, CDCl_3_) δ (ppm) 8.04 (s, 1H), 7.93 (s, 1H), 7.88 (s, 2H), 7.71 (s, 1H), 5.22–5.13 (m,5H), 5.06 (td, *J* = 9.7, 5.4 Hz, 5H), 4.99–4.95 (m, 8H), 4.92–4.87 (m, 2H), 4.82 (d, *J* = 12.6 Hz, 1H), 4.78 (d, *J* = 12.0 Hz, 1H), 4.73 (d, *J* = 12.5 Hz, 1H), 4.69–4.48 (m, 18H), 4.44 (d, *J* = 7.8 Hz, 1H), 4.26–4.21 (m, 11H), 4.16–4.10 (m, 5H), 4.05–3.89 (m, 5H), 3.78 (br s, 2H), 3.76–3.69 (m, 5H), 3.55–3.43 (m, 2H), 3.43–3.29 (m, 2H), 2.37–1.71 (m, 60H); ^13^C-NMR (150 MHz, CDCl_3_) δ (ppm) 170.6, 170.1, 169.4, 169.3, 169.2, 144.8, 144.7, 144.8, 144.5, 144.3, 124.8, 124.7, 124.6, 124.5, 124.2, 102.3, 100.6, 100.5, 100.4, 83.9, 81.7, , 74.4, 72.6, 72.5, 72.5, 72.48, 72.4, 71.9, 71.8, 70.8, 70.7, 68.8, 68.3, 68.2, 67.8, 67.7, 67.6, 66.4, 65.7, 65.7, 64.5, 62.8, 61.7, 61.72, 61.69, 53.4, 49.8, 49.76, 49.70, 20.7, 20.6, 20.5. ESI^+^-HRMS: [M + 2H]^2+^ calcd for C_101_H_139_N_15_O_56_, 1229.4246; found: 1229.4255.

**Synthesis of 11.** To a solution of **10** (40 mg, 0.016 mmol) in methanol-H_2_O (10:1) (3 mL) was added sodium methoxide in methanol (0.59 M) slowly to bring pH to 9. It was stirred for 18 h at room temperature. It was stirred with acidic resin (IR-120) to bring the pH around 7. The resin was filtered off and filtrate was evaporated, dissolved in water and lyophilized to give **11** as a fluffy solid (26 mg, 0.16 mmol, 98%). ^1^H-NMR (600 MHz, D_2_O) δ (ppm) 8.14 (s, 1H), 8.13 (s, 1H), 8.05 (s, 1H), 8.04 (s, 1H), 8.01 (s, 1H), 4.97 (d, *J* = 12.7 Hz, 1H), 4.83 (ddd, *J* = 12.5, 8.9, 3.4 Hz, 4H), 4.74–4.58 (m, 16H), 4.59–4.50 (m, 1H), 4.48–4.37 (m, 6H), 4.35–4.23 (m, 6H), 4.15–4.02 (m, 6H), 3.91–3.84 (m, 6H), 3.74–3.61 (m, 9H), 3.60–3.55 (m, 1H), 3.52 (dd, *J* = 15.8, 6.8 Hz, 1H), 3.49–3.37 (m, 12H), 3.36–3.27 (m, 5H), 3.26–3.16 (m, 5H); ^13^C-NMR (150 MHz, D_2_O + acetond-*d*_6_) δ (ppm) 144.4, 144.1, 144.1, 144.0, 126.6, 126.5, 126.3, 126.2, 126.1, 103.0, 102.9, 102. 8, 102.1, 83.4, 81.3, 77.1, 76.5, 76.2, 76.1, 73.9, 73.5, 73.4, 73.4, 72.6, 70.1, 70.1, 68.6, 68.5, 68.3, 66.0, 65.3, 65.2, 63.8, 63.0, 62.7, 61.3, 50.9, 50.8, 50.8, 28.8. ESI^+^-HRMS: [M + H]^+^ calcd for C_61_H_98_N_15_O_36_, 1616.6320; found: 1616.6293.

**Synthesis of 12. Procedure A:** To a solution of **11** (12 mg, 0.007 mmol) in dry DMF (3 mL) at 0 °C was added sodium hydride (60% in oil, 80 mg, 2 mmol). It was stirred at this temperature for 1 h and then at room temperature for another 2 h. The mixture was cooled back to 0 °C and allyl bromide (200 µL 2.3 mmol) was added slowly. After the addition the mixture was stirred at room temperature for 18 h. Saturated NH_4_Cl was added to quench the reaction. It was extracted with EtOAc (50 mL). The extract was washed with brine (5 mL), dried over MgSO_4_ and evaporated. The crude material was chromatographed over silica gel using hexane-EtOAc (3:2) and 5% to 10% methanol in DCM as eluents yielding perallylated product **12** as colorless oil (14 mg, 0.058 mmol, 77%).

**Procedure B:** Compound **12** could also be obtained by treating **5** (15 mg, 0.04 mmol) and **7** (135 mg, 0.33 mmol) under CuAAC reaction condition using Cu(I)·P(OEt)_3_ (8 mg, 0.02 mmol) as catalyst in toluene (2.4 mL) by irradiating under microwave at 70 °C for 4 h. Product **12** was isolated by column chromatography after usual work-up in 80% yield (78 mg, 0.032 mmol). ^1^H-NMR (300 MHz, CDCl_3_) δ (ppm) 8.08, 8.03, 7.98, 7.93 and 7.80 (5 × s, 5H), 5.98–5.72 (m, 20H), 5.29–5.06 (m, 39H), 4.99–4.75 (m, 8H), 4.71–4.53 (m, 13H), 4.43–4.41 (m, 2H), 4.35–4.19 (m, 24H), 4.13–3.96 (m, 29H), 3.76–3.58 (m, 12H), 3.49–3.47 (m, 3H), 3.35 (broad s, 17H), 3.18 (broad s, 6H); ^13^C-NMR (150 MHz, CDCl_3_) δ (ppm) 144.7, 135.2, 134.9, 134.8, 134.7, 134.6, 124.5, 117.2, 117.0, 116.9, 116.7, 103.2, 84.0, 81.2, 74.7, 74.4, 73.8, 73.5, 72.4, 68.7, 67.8, 50.1, 32.0, 29.7, 22.7. ESI^+^-HRMS: [M + 2H]^2+^ calcd for C_121_H_179_N_15_O_36_, 1209.6288; found: 1209.6329.

**Synthesis of 13.** A mixture of 2-azidoethyl 2,3,4,6-tetra-*O*-trimethylsilylpropargyl-β-d-glucopyranoside (**9**) (50 mg, 0.07 mmol), **5** (3.5 mg, 0.01 mmol), CuI·P(OEt)_3_ (3.5 mg, 0.01 mmol) and *N*,*N*-diisopropylethylamine (DIPEA) (30 µL, 0.17 mmol) in dry toluene (2 mL) was held at 65–70 °C with stirring for 20 h. The mixture was cooled and diluted with ethyl acetate (100 mL). It was washed with EDTA (3 × 5 mL), NaHCO_3_ solution (2 × 5 mL) and brine (5 mL). The extract was dried over MgSO_4_ and evaporated. The crude was purified by column chromatography over silica gel (hexane-ethyl acetate (1:1) and 2%–5% MeOH in toluene as eluents) yielding TMS-protected dendrimer **13** (32 mg, 0.009 mmol, 90%). ^1^H-NMR (300 MHz, CDCl_3_) δ (ppm) 8.14 (s, 1H), 8.09 (s, 1H), 7.99 (s, 2H), 7.84 (s, 1H), 5.03–4.74 (m, 9H), 4.73–4.36 (m, 35H), 4.36–4.11 (m, 30H), 4.07–3.85 (m, 11H), 3.84–3.55 (m, 9H), 3.54–3.29 (m, 24H), 0.17 (broad signal, 180H); ^13^C-NMR (150 MHz, CDCl_3_) δ (ppm) 144.8, 144.7, 144.5, 144.4, 144.2, 124. 7, 124.6, 124.4, 124.3, 124.0, 103.1, 103.0, 102.901, 102.0, 101.90, 101.80, 101.40, 91.2, 91.1, 91.0, 83.60, 81.1, 81.0, 80.9, 77.4, 75.9, 75.8, 74.5, 74.3, 68.9, 68.0, 67.9, 66.5, 65.9, 65.7, 64.6, 62.9, 61.0, 60.6, 60.2, 60.1, 60.0, 59.5, 50.0, 49.9, 49.8, 49.8, 30.9, 29.7, −0.1. FT-IR: ν_max_ (neat)/cm^−1^ 2177 (substituted propargyl).

**Synthesis of 14.** To a solution of **13** (49 mg, 0.013 mmol) in THF (2 mL) was added TBAF (1 M in THF, 300 µL, 0.3 mmol) and 4 drops of glacial acetic acid (adjusted to pH 7). The mixture was stirred at room temperature for 5 h. It was evaporated and dissolved in EtOAc (50 mL). The solution was washed with saturated NH_4_Cl solution (5 mL) and brine (5 mL) and dried over MgSO_4_. It was evaporated and passed through a column of silica gel (hexane-ethyl acetate (1:1) and 5%–10% MeOH in toluene as eluents) yielding perpropargylated dendrimer **14** as light yellow oil (26 mg, 0.011 mmol, 85%).

**Synthesis of 14 by direct alkylation:** To a solution of **11** (193 mg, 0.119 mmol) in DMF (15 mL) was added propargyl bromide (650 µL, 4.37 mmol). It was cooled to 0 °C. Four portions of NaH (60% in oil, 60 mg each, 1.5 mmol each) was added at an interval of one hour. After the additions, the ice-bath was removed and the mixture was stirred at room temperature for 18 h. The reaction was quenched with saturated NH_4_Cl solution and extracted with EtOAc (150 mL). The extract was washed with brine and dried over MgSO_4_. The solvent was removed and the crude product was passed through a column of silica gel (using hexane-EtOAc (1:1) and 0–10% MeOH in DCM as eluents) yielding mainly fully propargylated (230 mg) (slightly impure) with a small amount of partially propargylated (57 mg) product. Partially propargylated fraction was further propargylated using the above procedure with propargyl bromide (0.1 mL, 1.1 mmol) and three portions of sodium hydride (60% in oil, 10 mg each, 0.25 mmol each) in THF (4 mL). The product thus obtained was combined with slightly impure fraction (230 mg) and passed through a column of silica gel using hexane-EtOAc (1:1) and 4% methanol in DCM as eluents yielding perpropargylated product, **14** as light yellow oil (198 mg, 0.08 mmol, 70%). ^1^H-NMR (600 MHz, CDCl_3_) δ (ppm) 8.15 (s,1H), 8.11 (s, 1H), 8.05 (s, 1H), 8.01 (s, 1H), 7.89 (s, 1H), 5.01–4.97 (m, 3H), 4.93–4.88 (m, 2H), 4.82 (dd, *J* = 11.8, 8.9 Hz, 2H), 4.75 (d, *J* = 12.4 Hz, 1H), 4.71–4.56 (m, 14H), 4.54–4.37 (m, 25H), 4.35–4.10 (m, 36H), 4.04–3.97 (m, 6H), 3.86–3.79 (m, 6H), 3.78–3.71 (m, 8H), 3.54–3.48 (m, 8H), 3.47–3.29 (m, 20H), 2.60–2.40 (m, 20H); ^13^C-NMR (150 MHz, CDCl_3_) δ (ppm) 144.79, 144.72, 144.26, 128.97, 128.16, 125.23, 124.90, 124.84, 124.60, 102.98, 102.19, 84.01, 83.34, 75.01, 74.95, 74.50, 74.44, 73.98, 68.39, 67.96, 65.73, 64.64, 62.86, 60.19, 59.93, 59.92, 59.34, 49.88. FT-IR: ν_max_ (neat)/cm^−1^ 3283s (*br*, C≡CH), 2118 (≡CH). ESI^+^-HRMS: [M + 2H]^2+^ calcd for C_121_H_139_N_15_O_36_, 1189.4779; found: 1189.4764.

**Synthesis of 15.** A solution of perallylated intermediate **12** (8 mg, 0.003 mmol), mercaptoethanol (28 mg, 0.036 mmol, 120 eq.) and a catalytic amount of AIBN in dioxane (2.0 mL) was stirred at reflux for 1 h. The mixture was then diluted with H_2_O, dialysed against water over night, and lyophilized to yield polyol G_2_ (**15**) as light yellow oil (5.6 mg, 0.0014 mmol, 47%). ^1^H-NMR (600 MHz, D_2_O) δ (ppm) 8.20–8.04 (5H, m), 5.05–4.86 (7H, m), 4.74–4.58 (13H, m), 4.49–4.21 (13H, m), 4.17–3.97 (20H, m), 3.92–3.56 (73H, m), 3.54–3.23(26H, m), 3.21–2.92 (27H, m), 2.92–2.57 (47H, m), 2.56–2.41(5H, m), 2.20–2.01 (10H, broad signal), 1.99–1.81 (20H, broad signal), 1.75–1.57 (5H, broad signal). MALDI-TOF-MS: C_161_H_297_N_15_O_56_S_20_; exact mass cald: 3976.52678; found: ~4000 (broad).

**Synthesis of 16.** The solution of perallylated **12** (10 mg, 0.004 mmol), thioglycerol (43 µL, 0.48 mmol, 120 equiv.) and a catalytic amount of AIBN in dioxane (2.0 mL) was stirred under reflux for 1 h. The mixture was then diluted with H_2_O, dialysed against water overnight, and lyophilized to yield polyol G_2_ (**16**) as yellow light oil (11 mg, 0.0024 mmol, 60%). ^1^H-NMR (600 MHz, D_2_O) δ 8.18–8.00 (5H, m), 5.09–4.81 (6H, m), 4.68 (12H, broad signal), 4.51–4.22 (11H, m), 4.22–4.02 (11H, m), 4.02–3.19 (131H, m), 3.16–2.88 (21H, m), 2.88–2.38 (61H, m), 2.21–2.00 (7H, broad signal), 2.00–1.76 (24H, broad signal), 1.74–1.51 (8H, broad signal); ^13^C-NMR (150 MHz, D_2_O) δ (ppm) 145.8, 145.4, 126.5, 103.7, 84.9, 82.7, 75.2, 73.5, 72.9, 72.7, 72.1, 71.1, 70.7, 70.3, 69.2, 68.2, 67.0, 66.3, 56.4, 55.6, 51.7, 49.7, 49.4, 35.8, 30.7, 29.9, 29.8, 24.6, 24.4, 24.3, 23.7. MALDI-TOF-MS: C_181_ H_337_N_15_O_76_S_20_; exact mass cald: 4576.73807; found: 4555.334.

**Synthesis of 17.** Perpropargylated **14** (10 mg, 0.004 mmol) and 2,2-dimethoxy-2-phenylacetophenone (DMPA), (12 mg, 0.047 mmol) in DMF (200 µL) was added in a quartz photometer cell. 2-Mercaptoethanol (200 mg, 2.56 mmol) was added into the solution. The mixture was irradiated under 365 nm light for 4 h. The mixture was then diluted with H_2_O, dialysed against water overnight, and lyophilized to yield polyol G_2_ (**17**) as light yellow oil (13 mg, 0.002 mmol, 56%). ^1^H-NMR (600 MHz, MeOD) δ (ppm) 8.25–8.11(5H, m), 5.10–4.93 (7H, m), 4.84–4.59 (19H, m), 4.50–4.27 (15H, m), 4.24–3.99 (34H, m), 3.96–3.56 (108H, m), 3.55–3.40 (14H, m), 3.28–2.63 (137H, m); ^13^C-NMR (150 MHz, MeOD) δ (ppm) 127.1, 126.5, 105.6, 86.5, 84.1, 79.8, 77.5, 76.6, 76.2, 76.1, 75.2, 71.8, 70.0, 63.9, 63.5, 63.3, 57.3, 56.9, 52.6, 49.1, 48.8, 48.3, 37.3, 37.2, 37.0, 36.2, 36.0. MALDI-TOF-MS: C_201_H_377_N_15_O_76_S_40_; exact mass: 5496.49249; found: 5590.

**Synthesis of 18.** To a solution of perpropargylated compound **14** (10 mg, 0.004 mmol) and DMPA (12 mg, 0.047 mmol) in DMF (200 µL) in a quartz photometer cell was added thiolglycerol (400 mg, 3.70 mmol). The mixture was irradiated under 365 nm light for 6 h. The mixture was then diluted with H_2_O, dialysed against water over night, and lyophilized yield polyol G2 (**18**) as light yellow solid (20 mg, 0.003 mmol, 71%).^1^H-NMR (600 MHz, D_2_O) δ (ppm) 8.23–8.04 (5H, m), 5.11–4.89 (10H, m), 4.55–4.42 (8H, m), 4.40–4.28 (7H, m), 4.27–3.98 (26H, m), 3.95–3.31(171H, m), 3.30–3.12 (23H, m), 3.10–2.59 (114H, m); ^13^C-NMR (150 MHz, D_2_O) δ (ppm) 145.1, 144.7, 126.3, 126.1, 103.2, 100.6, 100.4, 84.4, 84.0, 82.4, 78.3, 75.2, 74.5, 73.4, 71.8, 71.6, 70.1, 68.5, 67.6, 67.5, 66. 5, 65.6, 51.1, 47.0, 46.7, 46.4, 36.11, 35.4, 34.8. MALDI-TOF-MS: C_241_H_457_N_15_O_116_S_40_; exact mass: 6696.91507; found: 6745.

**Synthesis of polyol 19.** A mixture of 2-azidoethyl β-D-glucopyranoside (**6**) (34 mg, 0.136 mmol), **14** (8 mg, 0.003 mmol) and CuI·P(OEt)_3_ (3 mg, 0.008 mmol) in dry DMF (1 mL) was held at 70 °C for 21 h. It was cooled and diluted with water. The mixture was dialysed (in a dialysis bag with 1200 cut-off) against water (with frequent change of water at the start) for 72 h. The dialysed material was lyophilized to give **19** (13 mg, 0.002 mmol, 53%). ^1^H-NMR (600 MHz, D_2_O) δ (ppm) 8.07–7.95 (m, 25H), 4.69–4.51 and 4.48–4.34 (m, 116H), 4.29–4.17 (m, 28H), 4.12–3.91 (m, 27H), 3.88–3.80 (m, 22H), 3.69–3.57 (m, 32H), 3.45–3.36, 3.33–3.27, 3.22–3.16 (m, 97H); ^13^C-NMR (150 MHz, D_2_O + acetone-*d*_6_) δ (ppm) 144.2, 126.4, 103.1, 83.4, 81.3, 76.6, 76.3, 74.0, 73.62 70.3, 68.6, 66.0, 65.5, 63.9, 61.4, 51.0. MALDI-TOF-MS: C_281_H_437_N_75_O_156_; exact mass: 7357.85677; found: 7519.337.

**Synthesis of 20.** A mixture of **14** (13 mg, 0.006 mmol), 2-azidoethyl 2,3,4,6-tetra-*O*-acetyl-β-d-glucopyranoside (**3**) (86 mg, 0.20 mmol) and CuI·P(OEt)_3_ (5.5 mg, 0.02 mmol) in dry DMF (1.3 mL) was stirred at 65–70 °C for 20 h. It was cooled and evaporated. The reaction mixture was diluted with EtOAc (100 mL), washed with EDTA (2 × 5 mL), NaHCO_3_ solution (2 × 5 mL) and brine, dried over MgSO_4_ and evaporated. The crude product was passed through a column of silica gel with hexane-EtOAc (1:1), 3% to 5% MeOH in toluene and 5% to 8% MeOH in DCM as eluents yielding **20** as colorless oil (39 mg, 0.004 mmol, 67%). ^1^H-NMR (600 MHz, CDCl_3_) δ (ppm) 8.13–7.61 (m, 25H), 5.20–5.14 (m, 22H), 5.09–5.04 (m, 19H), 4.99–4.82 (m, 37H), 4.80–4.47 (m, 95H), 4.39–4.33 (m, 9H), 4.31–4.17 (m, 44H), 4.17–4.08 (m, 20H), 4.07–3.97, 3.97–3.90 (m, 24 H), 3.81–3.68 (m, 30H), 3.53–3.44 (m, 10H), 3.41–3.26 (m, 15H), 2.07–1.91 (m, 240H); ^13^C-NMR (150 MHz, CDCl_3_) δ (ppm) 170.6, 170.1, 169.4, 169.3, 169.2, 144.5, 124.6, 103.4, 100.5, 100.4, 83.7, 81.7, 74.2, 72.6, 72.5, 71.8, 70.9, 70.8, 68.3, 67.7, 65.7, 64. 5, 61.8, 49.7, 29.7, 20.7, 20.6. MALDI-TOF MS: C_441_H_597_N_75_O_236_; exact mass: 10,718.70195; found: 10,747.228. See Supplementary Materials for GPC.

**Synthesis of 21.** A mixture of 2-azidoethyl 2,3,4,6-tetra-*O*-allyl-β-d-glucopyranoside (**7**) (85 mg, 0.20 mmol), **14** (13 mg, 0.006 mmol) and CuI·P(OEt)_3_ (5.5 mg, 0.02 mmol) in dry DMF (1.3 mL) was stirred at 65–70 °C for 20 h. The reaction mixture was evaporated and the residue was dissolved in EtOAc (100 mL). It was washed with EDTA (2 × 5 mL), saturated NaHCO_3_ solution (2 × 7 mL) and brine (5 mL), and dried over MgSO_4_. The solution was evaporated and passed through a column of silica gel with hexane-EtOAc (1:1) and 2% to 10% MeOH in DCM as eluents yielding **21** as colorless oil (33 mg, 0.003 mmol, 57%). ^1^H-NMR (600 MHz, CDCl_3_) δ (ppm) 8.14–7.70 (m, 25H), 6.09–5.65 (m, 80H), 5.42–5.01 (m, 159H), 5.01–4.49 (m, 100H), 4.37–4.18 (m, 107H), 4.15–3.86 (m, 129H), 3.77–3.53 (m, 58H), 3.46 (broad signal, 17H), 3.34 (broad signal, 69H), 3.21–3.12 (m, 22H); ^13^C-NMR (150 MHz, CDCl_3_) δ (ppm) 144.67, 144.33, 135.21, 135.16, 135.05, 134.96, 134.87, 134.78, 134.61, 134.57, 124.39, 124.00, 117.16, 116.92, 116.84, 116.63, 103.41, 103.16, 83.97, 81.23, 74.72, 74.32, 73.75, 73.43, 72.40, 68.70, 67.77, 66.42, 65.81, 64.51, 49.96, 29.66, 22.65. MALDI-TOF-MS: C_521_H_757_N_75_O_156_; exact mass: 10,560.36078; found: 10,632.893. See SI for GPC.

**Computational Methods.** The computational approach used for this study is the same recently adopted for the simulation of a variety of dendrimers in aqueous solution [26,27,28,29,30]. The simulation work was conducted using the AMBER 12 software (version 12, University of California, San Francisco, CA, USA, 2012) [31]. The molecular model for **16** has been parameterized according to the “general AMBER force field (GAFF)” [32].

Dendrimer **16** was immersed in a simulation box filled with explicit TIP3P water molecules [33]. After preliminary energy minimization, the system has been thermalized through a short 100 ps MD simulation in NVT (constant N: number of atoms, V: volume and T: temperature) periodic boundary conditions to reach the temperature of 37 °C (310 K). Then, dendrimer **16** has been equilibrated through 200 ns of MD simulations NPT in periodic boundary condition (constant N: number of atoms, P: pressure and T: temperature during the run) at 37 °C of temperature and 1 atm of pressure. During this time, dendrimer **16** reached the equilibrium with good stability, as demonstrated by the root mean square displacement (RMSD), radius of gyration (R_g_) and enthalpy (H) data reported in the SI. For the MD run, a 2 femtoseconds time step was used, the Langevin thermostat, a 8 Å cutoff, the particle mesh Ewald [34] (PME) for long-range electrostatics, and the SHAKE algorithm to treat bonds involving Hydrogen atoms [35] Enthalpy H was calculated through the MMPBSA approach [36,37] according to the same procedure followed previously [26,27,28,29,30]. Namely, H is the sum of the total gas-phase in vacuo non-bonded energy (Egas) and a solvation term (Gsol = G_PB_ + G_NP_) [38] The polar component of G_PB_ was evaluated using the Poisson-Boltzmann [39] (PB) term while the non-polar contribution to the solvation energy was calculated as G_NP_ = a (SASA) + b, in which a = 0.00542 kcal/Å^2^, b = 0.92 kcal/mol, and the SASA (solvent accessible surface area) was estimated with the MOLSURF program included in AMBER 12 [40]. The last 100 ns of the MD trajectories were considered as representative of the equilibrium condition for dendrimer **16** and used for the analysis of the structural parameters reported in Table S1.

## 4. Conclusions

An efficient synthesis of dense and chiral dendritic polyols using sugars as multivalent nanosynthons (glyconanosynthons) at the core and at the repeating units was established. The click reactions worked best with a catalytic amount of the copper (I) organic catalyst CuI·P(OEt)_3_. The high yielding synthetic strategy happens to be very versatile, as it permitted access to the desired functionalized materials by many different alternatives, depending on the surface group functionality of the building blocks chosen. In addition, the alkenyl and alkynyl surface groups could be conveniently transformed into dendritic polyols using thiol-ene or thyol-yne reactions using either thioethanol or thioglycerol, respectively. Hence, the number of OH-groups that could be added can be readily doubled. Furthermore, in principle, the allyl end groups of **21** can be further transformed by an additional layer of protected sugar derivative to reach the **G_3_** level via a thiol-ene reaction to afford dendrimers with 160 hydroxyl groups using thioglycerol and with 320 hydroxyl groups using 2-thiolethyl β-d-glucopyranoside, as shown previously [12,13]. It is also possible to add cysteamine to the double bond of **21** to construct cationic dendrimers, which may find application as gene transfection agents. Moreover, the synthesis is able to generate dendrimers with defined stereochemistry and with more rigidity compared to the existing ones. For instance, polyol **16** had a diameter of 2 nm, as calculated using molecular dynamic simulations. The strategy described herein can thus provide dendrimers having a large number of surface functionalities at very low generation efficiently and rapidly, hence simplifying the entire process of making complex architectures. Work is also in progress with the cationic equivalents of these structures as gene transfecting agents [13] together with their potential as chiral catalysts.

## Supplementary Materials

The following are available online at http://www.mdpi.com/1420-3049/21/4/448/s1: NMR, MS, IR spectra, GPCs, Table S1 and Figure S1.

## References

[1] Caminade A.-M., Ouali A., Laurent R., Olivier Turrin C. (2015). Majoral, J.-P. The dendritic effect illustrated with phosphorus dendrimers. Chem. Soc. Rev..

[2] Sharma A., Kakkar A. (2015). Designing Dendrimer and Miktoarm Polymer Based Multi-Tasking Nanocarriers for Efficient Medical Therapy. Molecules.

[3] Tomalia D.A., Naylor A.M., Goddard W.A. (1990). Starburst Dendrimers: Molecular-Level Control of Size, Shape, Surface Chemistry, Topology, and Flexibility from Atoms to Macroscopic Matter. Angew. Chem. Int. Ed..

[4] Mignani S., El Kazzouli S., Bousmina M., Majoral J.P. (2013). Expand classical drug administration ways by emerging routes using dendrimer drug delivery systems: A concise overview. Adv. Drug Deliv. Rev..

[5] Roy R., Shiao T.C. (2015). Glyconanosynthons as powerful scaffolds andbuilding blocks for the rapid construction of multifaceted, dense and chiral dendrimers. Chem. Soc. Rev..

[6] Sowinska M., Urbanczyk-Lipkowska Z. (2014). Advances in the chemistry of dendrimers. New J. Chem..

[7] Walter M.V., Malkoch M. (2012). Simplifying the synthesis of dendrimers: Accelerated approaches. Chem. Soc. Rev..

[8] Astruc D., Boisselier E., Ornelas C. (2010). Dendrimers designed for functions: From physical, photophysical, and supramolecular properties to applications in sensing, catalysis, molecular electronics, photonics, and nanomedicine. Chem. Rev..

[9] Chabre Y.M., Roy R. (2010). Design and creativity in synthesis of multivalent neoglycoconjugates. Adv. Carbohydr. Chem. Biochem..

[10] Chabre Y.M., Roy R. (2008). Recent trends in glycodendrimer syntheses and apllications. Curr. Top. Med. Chem..

[11] Chabre Y.M., Roy R. (2013). Multivalent glycoconjugate syntheses and applications using aromatic scaffolds. Chem. Soc. Rev..

[12] Sharma R., Kottari N., Chabre Y.M., Rej R., Saadeh N.K., Roy R. (2014). “Onion peel” dendrimers: A straightforward synthetic approach towards highly diversified architectures. Polym. Chem..

[13] Sharma R., Zhang I., Shiao T.C., Pavan G.M., Maysinger D., Roy R. (2016). Low generation polyamine dendrimers bearing flexible tetraethylene glycol as nanocarriers for plasmids and siRNA. Nanoscale.

[14] Shiao T.C., Giguère D., Wisse P., Roy R. (2014). Synthesis of Phenyl 2,3,4,6-Tetra-*O*-acetyl-1-thio-β-d-galactopyranoside. Carbohydr. Chem. Proven Synth. Methods.

[15] Mereyala H.B., Gurrala S.R. (1998). A highly diastereoselective, practical synthesis of allyl, propargyl 2,3,4,6-tetra-*O*-acetyl-β-d-gluco,β-d-galactopyranosides and allyl, propargyl heptaacetyl-β-d-lactosides. Carbohydr. Res..

[16] Ortega-Munoz M., Morales-Sanfrutos J., Perez-Balderas F., Hernandez-Mateo F., Giron-Gonzalez M.D., Sevillano-Tripero N., Salto-Gonzalez R., Santoyo-Gonzalez F. (2007). Click multivalent neoglycoconjugates as synthetic activators in cell adhesion and stimulation of monocyte/macrophage cell lines. Org. Biomol. Chem..

[17] Quagliotto P., Viscardi G., Barolo C., D’Angelo D., Barni E., Compari C., Duce E., Fisicaro E. (2005). Synthesis and Properties of New Glucocationic Surfactants: Model Structures for Marking Cationic Surfactants with Carbohydrates. J. Org. Chem..

[18] Paterson S., Clark J., Stubbs K.A., Chirila T.V., Baker M.V. (2011). Carbohydrate-based cross-linking agents: Potential use in hydrogels. J. Polym. Sci. A Polym. Chem..

[19] Park S., Shin I. (2007). Carbohydrate Microarrays for Assaying Galactosyltransferase Activity. Org. Lett..

[20] Kottari N., Chabre Y.M., Shiao T.C., Rej R., Roy R. (2014). Efficient and accelerated growth of multifunctional dendrimers using orthogonal thiol-ene and S_N_2 reactions. Chem. Commun..

[21] Fleischmann S., Komber H., Voit B. (2008). Diblock Copolymers as Scaffolds for Efficient Functionalization via Click Chemistry. Macromolecules.

[22] Binauld S., Hawker C.J., Fleury E., Drockenmuller E. (2009). A Modular Approach to Functionalized and Expanded Crown Ether Based Macrocycles Using Click Chemistry. Angew. Chem. Int. Ed..

[23] Grayson S.M., Jayaraman M., Fréchet J.M.J. (1999). Convergent synthesis and “surface” functionalization of a dendritic analog of poly(ethylene glycol). Chem. Commun..

[24] Papp I., Dernedde J., Enders S., Riese S.B., Shiao T.C., Roy R., Haag R. (2011). Multivalent Presentation of Mannose on Hyperbranched Polyglycerol and their Interaction with Concanavalin A Lectin. Chembiochem.

[25] Goyard D., Shiao T.C., Giguère D., Roy R. (2015). Thiol-yne coupling between propargyl 2,3,4,6-tetra-*O*-acetyl-β-d-glucopyranoside and dodecanethiol. Access to mono- and bis-adducts. Carbohydr. Chem. Proven Synth. Methods.

[26] Pavan G.M. (2014). Modeling the interaction between dendrimers and nucleic acids: A molecular perspective through hierarchical scales. Chem. Med. Chem..

[27] Enciso A.E., Garzoni M., Pavan G.M., Simanek E.E. (2015). Influence of linker groups on the solubility of triazine dendrimers. New J. Chem..

[28] Pavan G.M., Barducci A., Albertazzi L., Parrinello M. (2013). Combining metadynamics simulation and experiments to characterize dendrimers in solution. Soft Matter.

[29] Liu Y., Bryantsev V.S., Diallo M.S., Goddard W.A. (2009). PAMAM dendrimers undergo pH responsive conformational changes without swelling. J. Am. Chem. Soc..

[30] Caminade A.M., Fruchon S., Turrin C.O., Poupot M., Ouali A., Maraval A., Garzoni M., Maly M., Furer V., Kovalenko V. (2015). The key role of the scaffold on the efficiency of dendrimer nanodrugs. Nat. Commun..

[31] Case D.A., Darden T.A., Cheatham T.E., Simmerling C.L., Wang J., Duke R.E., Luo R., Walker R.C., Zhang W., Merz K.M. (2012). AMBER 12.

[32] Wang J., Wolf R.M., Caldwell J.W., Kollman P.A., Case D.A. (2004). Development and testing of a general Amber force field. J. Comput. Chem..

[33] Jorgensen W.L., Chandrasekhar J., Madura J.D., Impey R.W., Klein M.L. (1983). Comparison of simple potential functions for simulating liquid water. J. Chem. Phys..

[34] Darden T., York D., Pedersen L. (1993). Particle mesh Ewald: An *N*·log(*N*) method for Ewald sums in large systems. J. Chem. Phys..

[35] Kräutler V., van Gunsteren W.F., Hunenberger P.H. (2001). A fast SHAKE algorithm to solve distance constraint equations for small molecules in molecular dynamics simulations. J. Comput. Chem..

[36] Kollman P.A., Massova I., Reyes C., Kuhn B., Huo S.H., Chong L., Lee M., Lee T., Duan Y., Wang W. (2000). Calculating structures and free energies of complex molecules: Combining molecular mechanics and continuum models. Acc. Chem. Res..

[37] Srinivasan J., Cheatham T.E., Cieplak P., Kollman P.A., Case D.A. (1998). Continuum Solvent Studies of the Stability of DNA, RNA, and Phosphoramidate-DNA Helices. J. Am. Chem. Soc..

[38] Jayaram B., Sprous D., Beveridge D.L. (1998). Solvation free energy of biomacromolecules: Parameters for a modified generalized born model consistent with the AMBER force field. J. Phys. Chem..

[39] Sitkoff D., Sharp K.A., Honig B. (1994). Accurate calculation of hydration free energies using macroscopic solvent models. J. Phys. Chem..

[40] Connolly M.L. (1983). Analytical molecular surface calculation. J. Appl. Cryst..

