# Supplementary material for: Synthesis of Dense and Chiral Dendritic Polyols Using Glyconanosynthon Scaffolds
Source: Molecules. 2016 Apr 4;21(4):448. doi: 10.3390/molecules21040448 (PMC6274151; doi:10.3390/molecules21040448)
Supplement: Supplementary file 1 [file molecules-21-00448-s001.pdf]

Supplementary Information: Synthesis of Dense and Chiral Dendritic Polyols Using Glyconanosynthon Scaffolds

Tze Chieh Shiao, Rabindra Rej, Mariecka Rose, Giovanni M. Pavan and René Roy

1. Characterizations (^{1}H, ^{13}C and COSY NMR; HRMS and GPC)

1.1. Propargyl 2,3,4,6-tetra-O-propargyl-β-D-glucopyranoside (**5**)

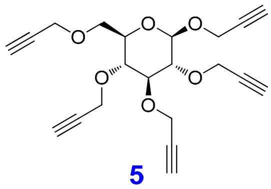

5

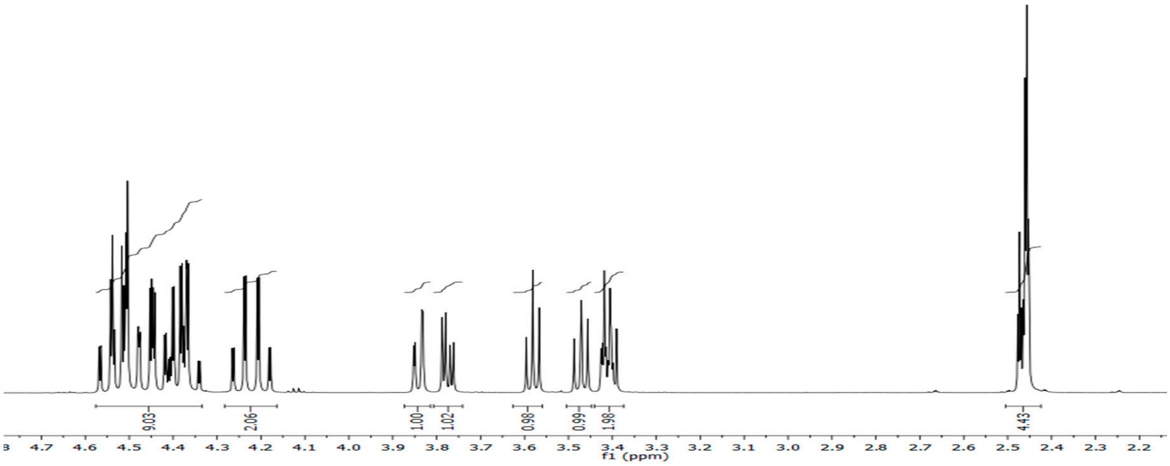

Figure S1. ^{1}H-NMR spectrum of Propargyl 2,3,4,6-tetra-*O*-propargyl-β-D-glucopyranoside (**5**).

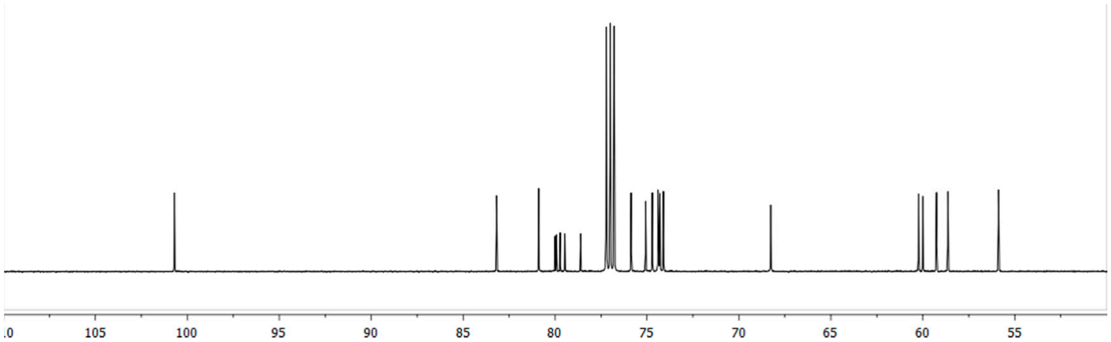

Figure S2. ^{13}C-NMR spectrum of Propargyl 2,3,4,6-tetra-*O*-propargyl-β-D-glucopyranoside (**5**).

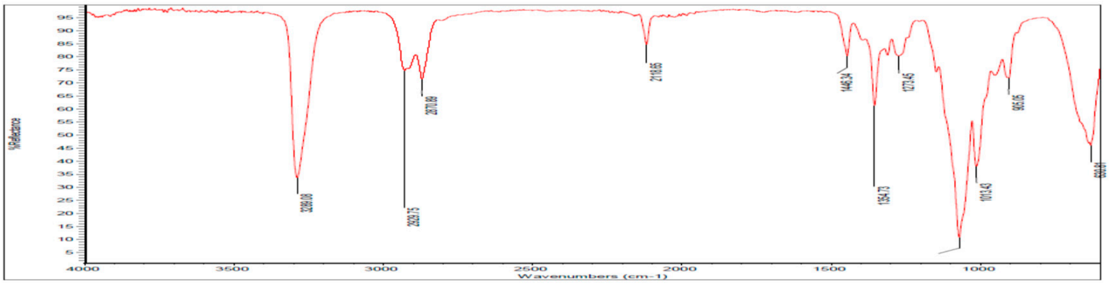

Figure S3. IR spectrum of Propargyl 2,3,4,6-tetra-*O*-propargyl-β-D-glucopyranoside (**5**).

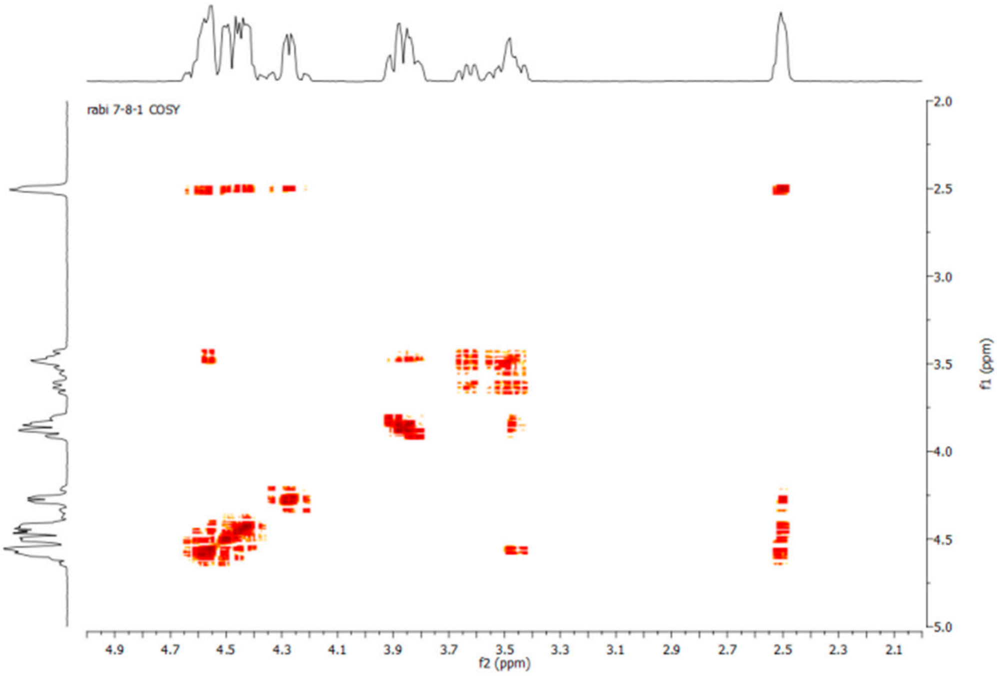

Figure S4. ^{1}H-^{1}H COSY-NMR of **5**.

1.2. *2-Azidoethyl 2,3,4,6-tetra-O-propargyl-β-D-glucopyranoside* (8)

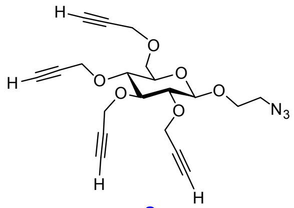

8

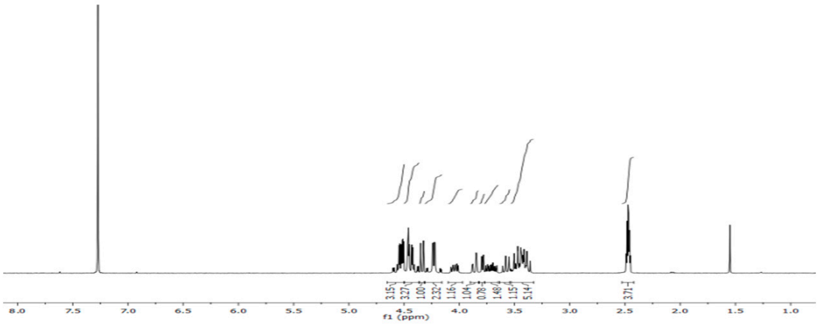

Figure S5. ^{1}H-NMR spectrum of 2-azidoethyl 2,3,4,6-tetra-*O*-propargyl-β-D-glucopyranoside (**8**).

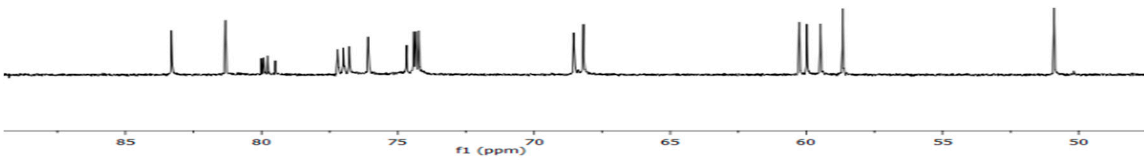

Figure S6. ^{13}C-NMR spectrum of 2-azidoethyl 2,3,4,6-tetra-*O*-propargyl-β-D-glucopyranoside (**8**).

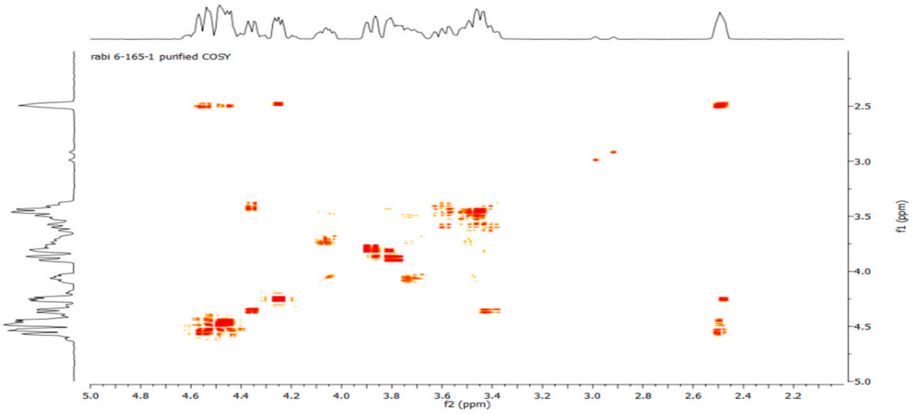

Figure S7. ^{1}H-^{1}H COSY-NMR of 2-azidoethyl 2,3,4,6-tetra-*O*-propargyl-β-D-glucopyranoside (**8**).

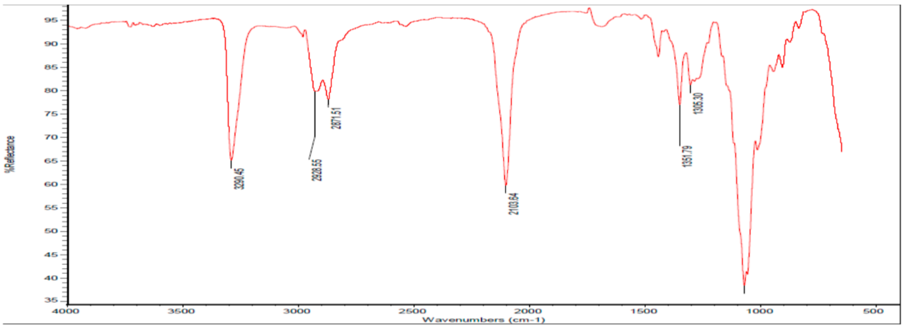

Figure S8. IR spectrum of **8**.

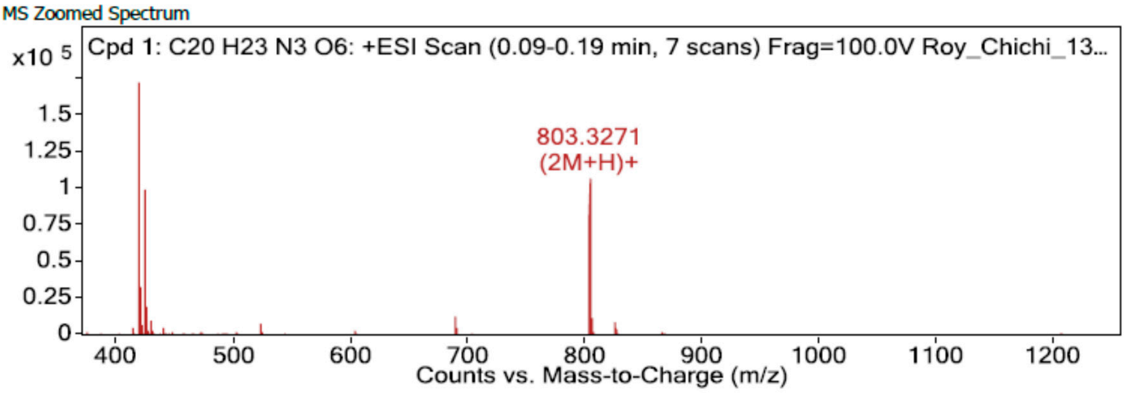

MS Spectrum Peak List

m/z	*Calc m/z*	Diff(ppm)	z	Abund	Formula	Ion
259.0962				177867		
259.2954				8441		
260.099				23956		
261.1043				3324		
402.1677	402.166	4.25	1	633	C20 H24 N3 O6	(M+H)+
406.1402	406.1373	7.04	1	54	C20 H21 N3 Na O5	(M+Na)+[-H2O]
419.1915	419.1925	-2.33	1	176460	C20 H27 N4 O6	(M+NH4)+
420.1945	420.1955	-2.31	1	34190	C20 H27 N4 O6	(M+NH4)+
424.1469	424.1479	-2.31	1	99987	C20 H23 N3 Na O6	(M+Na)+
425.1498	425.151	-2.91	1	19671	C20 H23 N3 Na O6	(M+Na)+
426.1521	426.1535	-3.21	1	2934	C20 H23 N3 Na O6	(M+Na)+
440.1214	440.1218	-1.06	1	4610	C20 H23 K N3 O6	(M+K)+
803.3271	803.3246	3.05	1	107356	C40 H47 N6 O12	(2M+H)+
804.3304	804.3277	3.32	1	46122	C40 H47 N6 O12	(2M+H)+
805.3335	805.3305	3.81	1	11151	C40 H47 N6 O12	(2M+H)+
825.3062	825.3066	-0.51	1	8644	C40 H46 N6 Na O12	(2M+Na)+
826.3086	826.3097	-1.32	1	3642	C40 H46 N6 Na O12	(2M+Na)+
841.2846	841.2805	4.82	1	372	C40 H46 K N6 O12	(2M+K)+
1204.4871	1204.4833	3.12	1	810	C60 H70 N9 O18	(3M+H)+
1226.4686	1226.4653	2.72	1	82	C60 H69 N9 Na O18	(3M+Na)+

Figure S9. High resolution mass spectrum of **8**.

*1.3. 2-Azidoethyl 2,3,4,6-tetra-O-trimethylsilylpropargyl-β-*D*-glucopyranoside* (**9**)

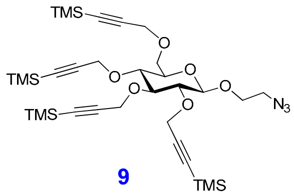

9

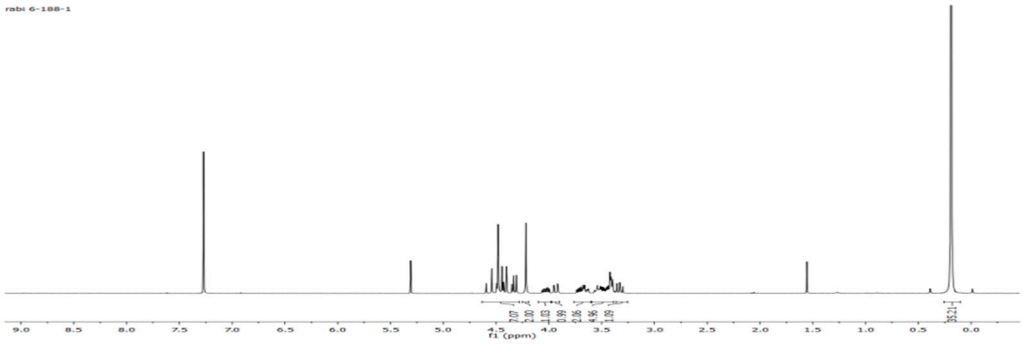

Figure S10. ^{1}H-NMR spectrum of **9**.

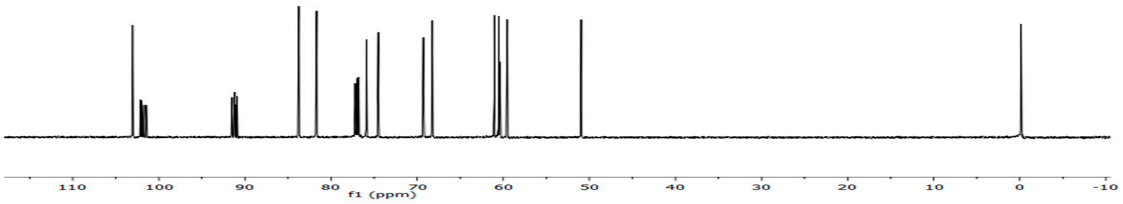

Figure S11. ^{13}C-NMR spectrum of **9**.

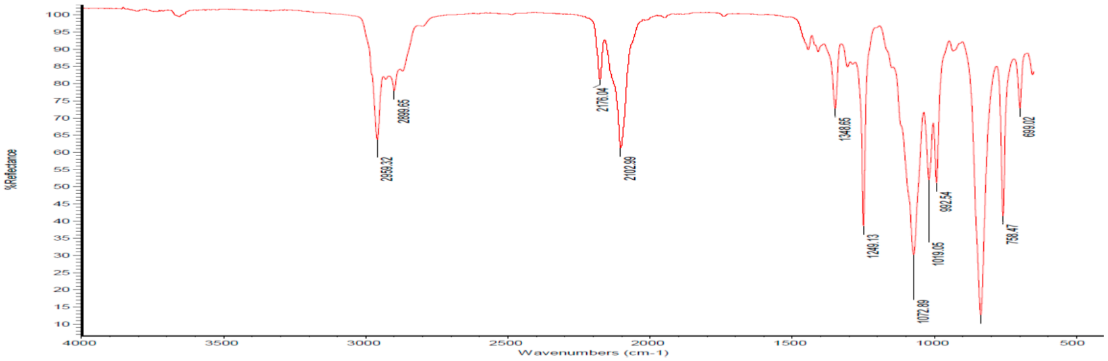

Figure S12. IR spectrum of **9**.

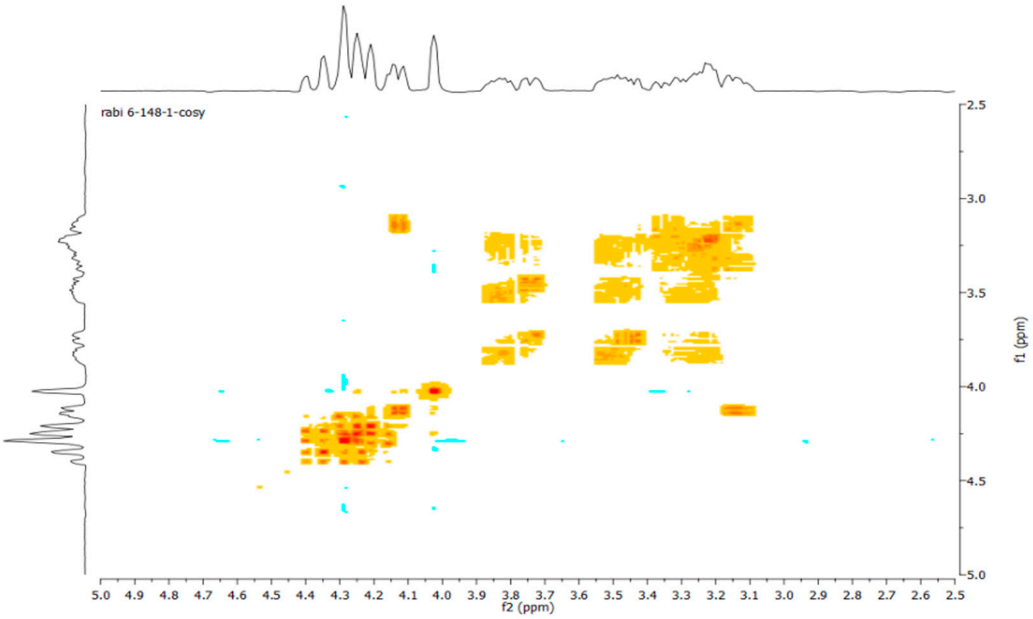

Figure S13. ^{1}H-^{1}H COSY-NMR of **9**.

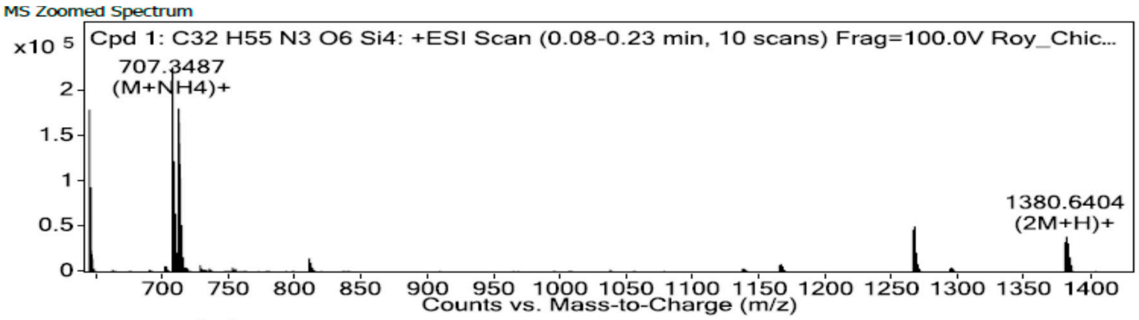

MS Spectrum Peak List

m/z	*Calc m/z*	Diff(ppm)	z	Abund	Formula	Ion
672.3154	672.3135	2.88	1	86	C32 H54 N3 O5 Si4	(M+H)+[-H2O]
690.3209	690.3241	-4.55	1	2500	C32 H56 N3 O6 Si4	(M+H)+
707.3487	707.3506	-2.75		239800	C32 H59 N4 O6 Si4	(M+NH4)+
707.6732				11424		
708.3501	708.3525	-3.35		129254	C32 H59 N4 O6 Si4	(M+NH4)+
709.3476	709.3514	-5.33		65366	C32 H59 N4 O6 Si4	(M+NH4)+
709.7971				7341		
710.344	710.3519	-11.13		21520	C32 H59 N4 O6 Si4	(M+NH4)+
711.341	711.3514	-14.67		6060	C32 H59 N4 O6 Si4	(M+NH4)+
712.3046	712.306	-2.04		192334	C32 H55 N3 Na O6 Si4	(M+Na)+
713.3073	713.3079	-0.86	1	106924	C32 H55 N3 Na O6 Si4	(M+Na)+
714.3069	714.3068	0.06	1	51427	C32 H55 N3 Na O6 Si4	(M+Na)+
715.3112	715.3073	5.38	1	16102	C32 H55 N3 Na O6 Si4	(M+Na)+
728.2773	728.28	-3.62	1	8165	C32 H55 K N3 O6 Si4	(M+K)+
1379.639	1379.6409	-1.34	1	33803	C64 H111 N6 O12 Si8	(2M+H)+
1380.6404	1380.6428	-1.72	1	39768	C64 H111 N6 O12 Si8	(2M+H)+
1381.6403	1381.6427	-1.74	1	31363	C64 H111 N6 O12 Si8	(2M+H)+
1382.6403	1382.6431	-2	1	17332	C64 H111 N6 O12 Si8	(2M+H)+
1383.6406	1383.643	-1.76	1	7801	C64 H111 N6 O12 Si8	(2M+H)+
1401.6216	1401.6228	-0.83	1	606	C64 H110 N6 Na O12 Si8	(2M+Na)+

Figure S14. High resolution mass spectrum of **9**.

1.4. Dendrimer **10**

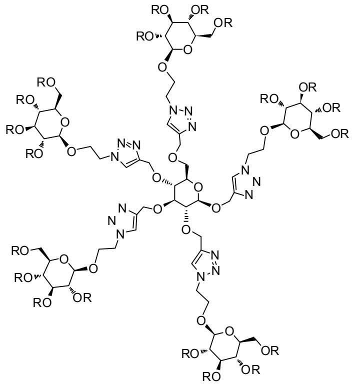

10 R = Ac

G_1 = 20-OAc

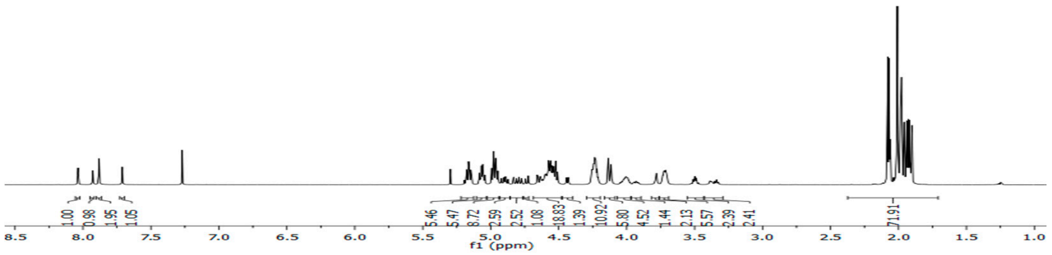

Figure S15. ^{1}H-NMR spectrum of **10**.

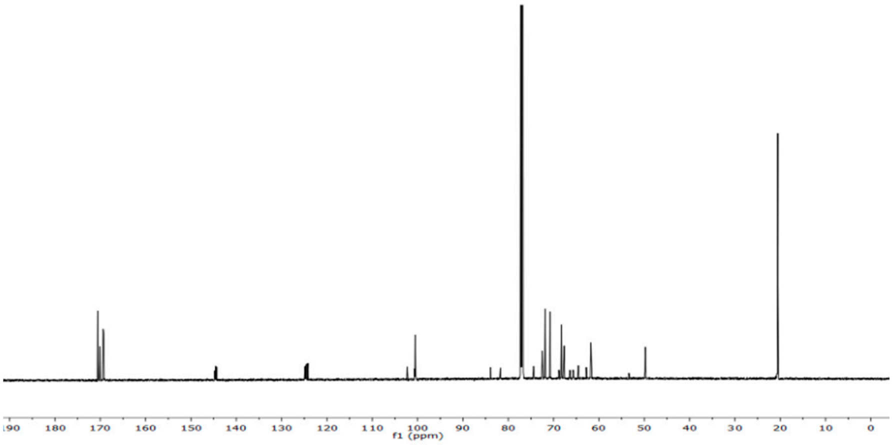

Figure S16. ^{13}C-NMR spectrum of **10**.

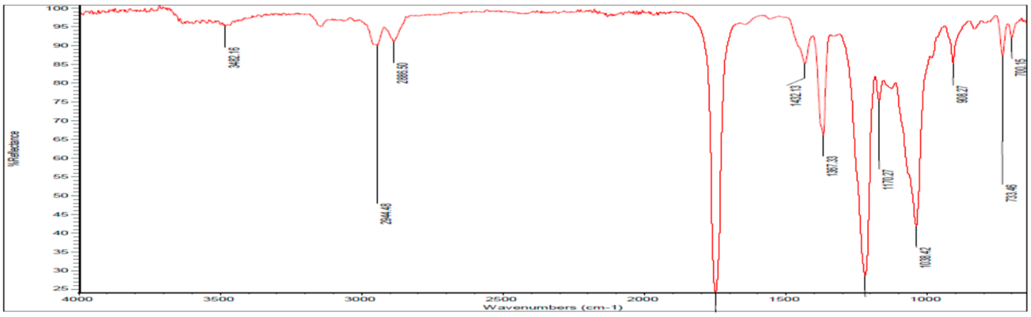

Figure S17. IR spectrum of **10**.

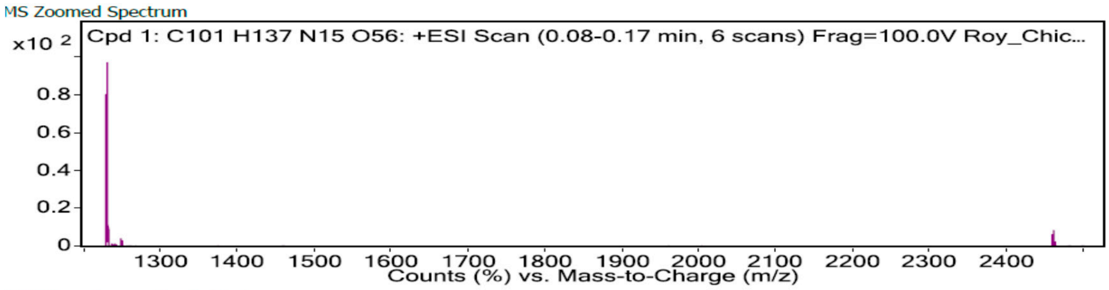

MS Spectrum Peak List

m/z	*Calc m/z*	Diff(ppm)	z	Abund	Formula	Ion
1228.9257	1228.924	1.44		304717	C101 H139 N15 O56	(M+2H)+2
1229.4246	1229.4255	-0.77		376627	C101 H139 N15 O56	(M+2H)+2
1229.9236	1229.9269	-2.74		264344	C101 H139 N15 O56	(M+2H)+2
1230.4226	1230.4283	-4.67		134676	C101 H139 N15 O56	(M+2H)+2
1230.9226	1230.9296	-5.73		55458	C101 H139 N15 O56	(M+2H)+2
1231.4221	1231.4309	-7.17		21270	C101 H139 N15 O56	(M+2H)+2
1231.9156	1231.9322	-13.46		9019	C101 H139 N15 O56	(M+2H)+2
1232.4072	1232.4335	-21.29		4364	C101 H139 N15 O56	(M+2H)+2
1232.6882				2781		
1232.896				2621		
2456.8347	2456.8406	-2.43	1	25699	C101 H138 N15 O56	(M+H)+
2457.8385	2457.8438	-2.13	1	32233	C101 H138 N15 O56	(M+H)+
2458.8391	2458.8466	-3.06	1	21581	C101 H138 N15 O56	(M+H)+
2459.8425	2459.8493	-2.75	1	9956	C101 H138 N15 O56	(M+H)+
2460.8482	2460.852	-1.52	1	3769	C101 H138 N15 O56	(M+H)+
2477.7929	2477.7891	1.56	1	492	C101 H135 K N15 O55	(M+K)+[-H2O]
2478.8307	2478.8226	3.29	1	2104	C101 H137 N15 Na O56	(M+Na)+
2479.834	2479.8257	3.37	1	2213	C101 H137 N15 Na O56	(M+Na)+
2494.7985	2494.7965	0.8	1	2379	C101 H137 K N15 O56	(M+K)+
2495.798	2495.7996	-0.67	1	2833	C101 H137 K N15 O56	(M+K)+

Figure S18. High resolution mass spectrum of (**10**).

1.5. Dendrimer **11**

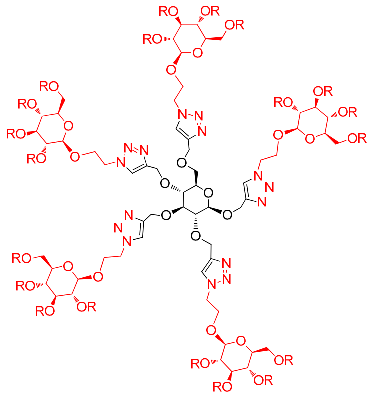

11 R = H

G_1 = 20-OH

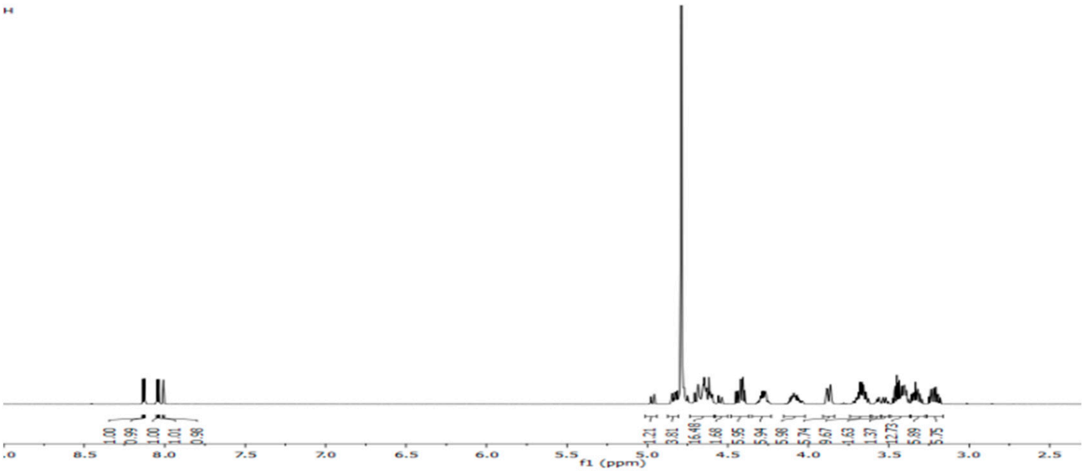

Figure S19. ^{1}H-NMR spectrum of **11**.

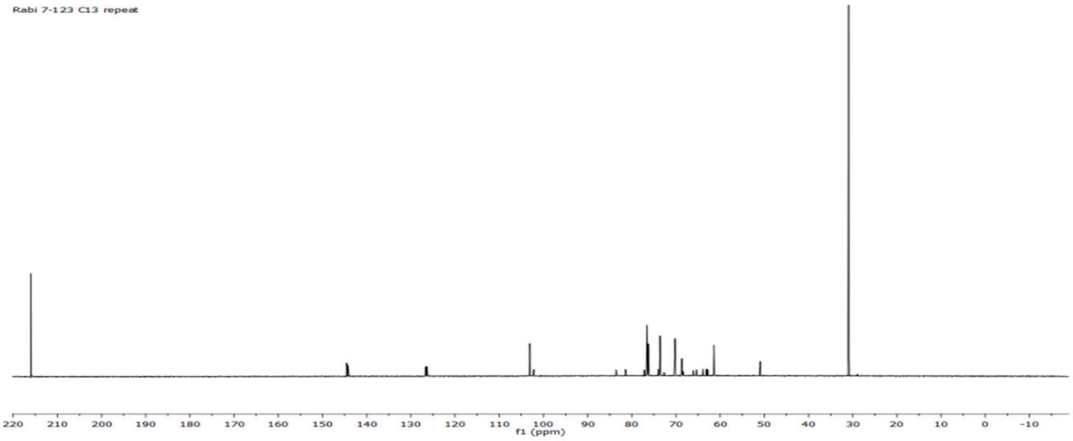

Figure S20. ^{13}C-NMR spectrum of **11**.

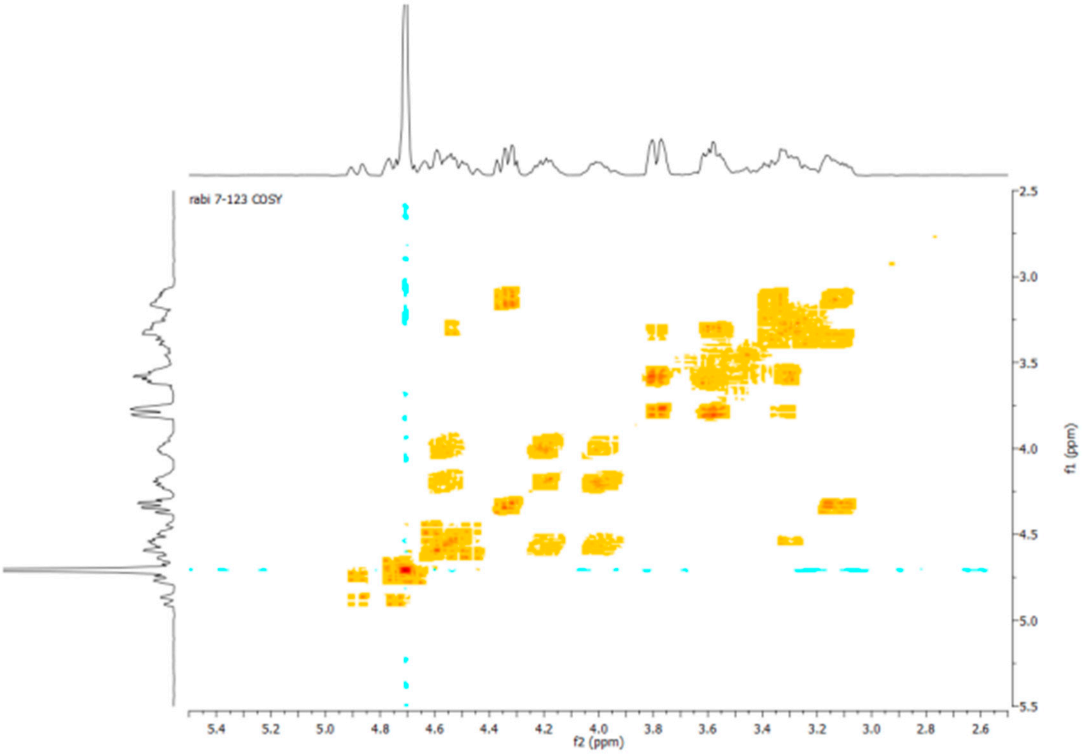

Figure S21. ^{1}H-^{1}H COSY-NMR of **11**.

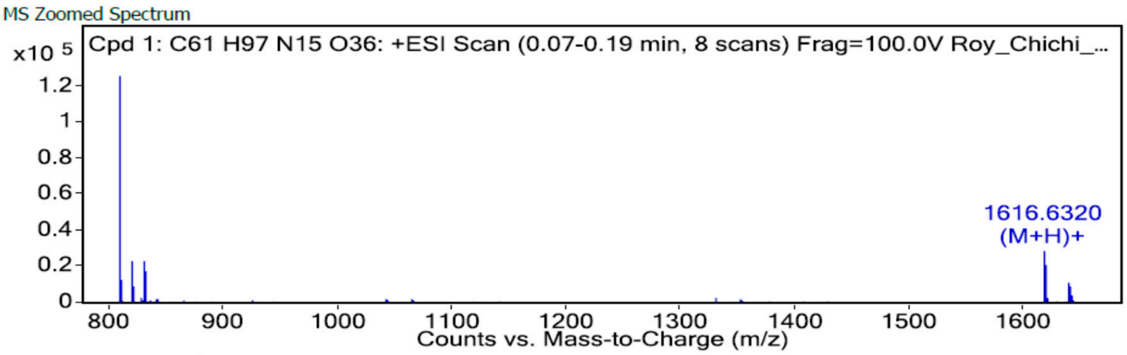

MS Spectrum Peak List

m/z	*Calc m/z*	Diff(ppm)	z	Abund	Formula	Ion
808.8206	808.8183	2.85		126172	C61 H99 N15 O36	(M+2H)+2
809.3219	809.3198	2.67		90605	C61 H99 N15 O36	(M+2H)+2
809.8235	809.8211	3.05		40449	C61 H99 N15 O36	(M+2H)+2
810.3248	810.3223	3.08		13374	C61 H99 N15 O36	(M+2H)+2
830.8022	830.8003	2.32	2	23375	C61 H97 N15 Na2 O36	(M+2Na)+2
831.3041	831.3017	2.84	2	17444	C61 H97 N15 Na2 O36	(M+2Na)+2
1616.632	1616.6293	1.67	1	29076	C61 H98 N15 O36	(M+H)+
1617.6345	1617.6323	1.34	1	20960	C61 H98 N15 O36	(M+H)+
1638.6062	1638.6113	-3.11	1	11885	C61 H97 N15 Na O36	(M+Na)+
1654.5751	1654.5852	-6.1	1	202	C61 H97 K N15 O36	(M+K)+

Figure S22. High resolution mass spectrum of **11**.

1.6. Dendrimer **12**

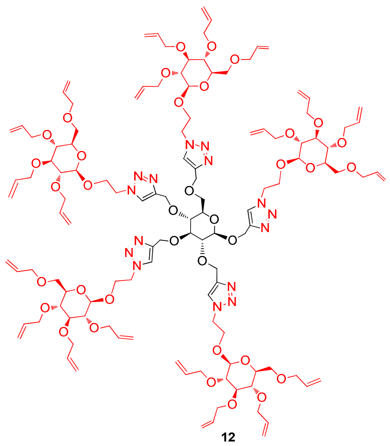

12

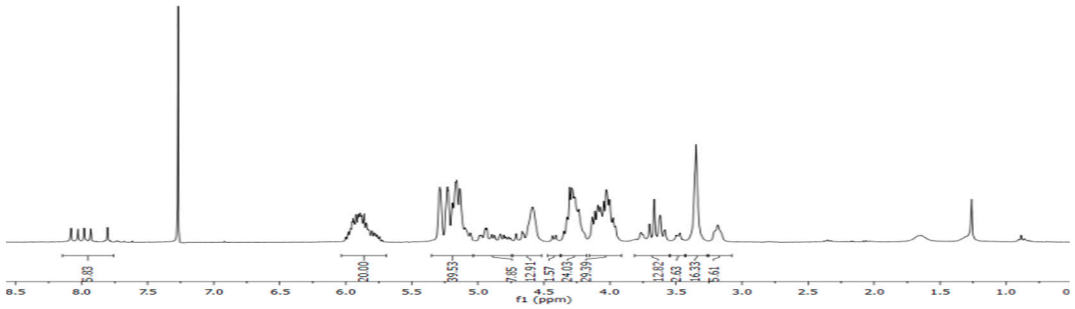

Figure S23. ^{1}H-NMR spectrum of **12** obtained by direct allylation.

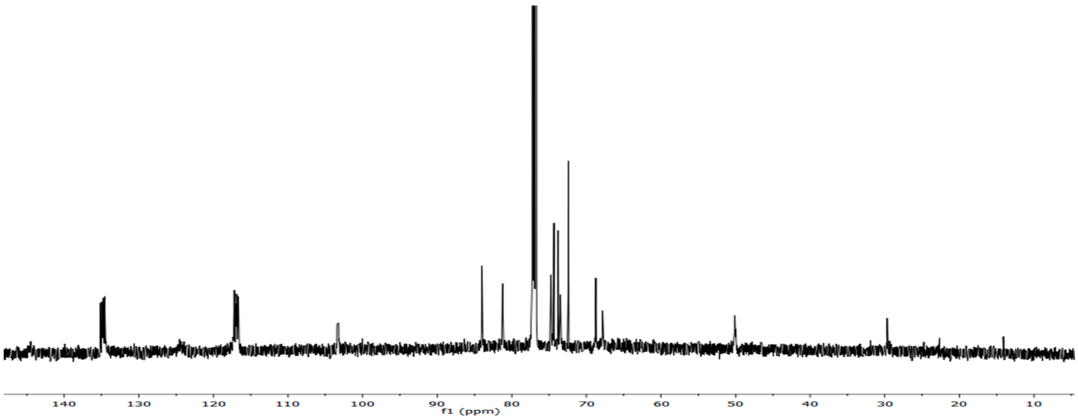

Figure S24. ^{13}C-NMR spectrum of **12**.

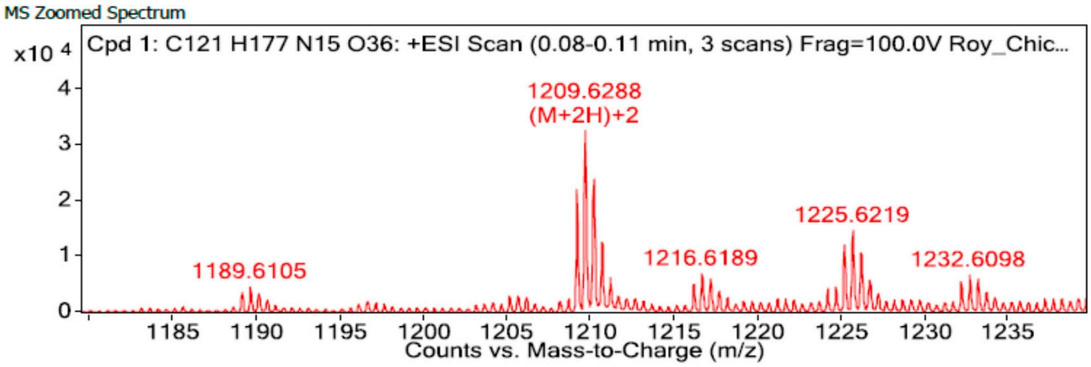

MS Spectrum Peak List

m/z	*Calc m/z*	Diff(ppm)	z	Abund	Formula	Ion
265.1448			1	12905		
506.2815			1	42318		
507.2846			1	11187		
1209.1281			2	22435		
1209.6288	1209.6329	-3.35	2	33404	C121 H179 N15 O36	(M+2H)+2
1210.1283	1210.1344	-5.05	2	24068	C121 H179 N15 O36	(M+2H)+2
1210.6266	1210.6359	-7.63	2	13130	C121 H179 N15 O36	(M+2H)+2
1225.1218			2	12518		
1225.6219			2	14930		
1226.1234			2	11121		

--- End Of Report ---

Figure S25. High resolution mass spectrum of **12**.

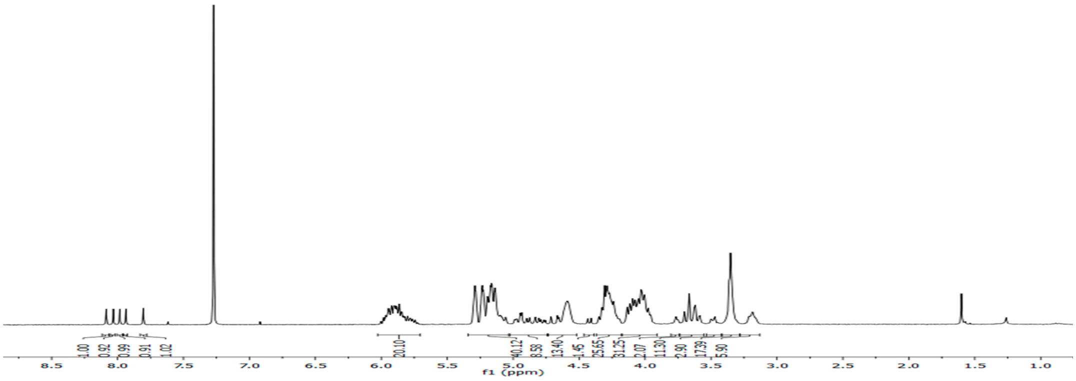

Figure S26. ^{1}H-NMR spectrum of **12** obtained from Click reaction.

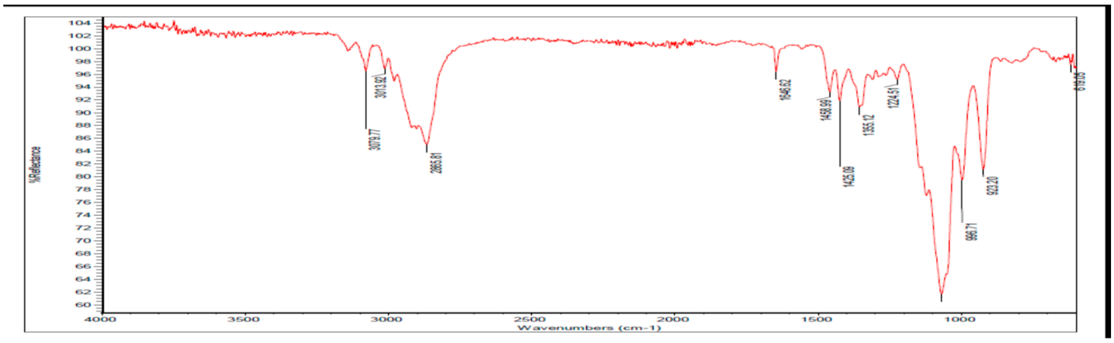

Figure S27. IR spectrum of of **12** obtained from Click reaction.

1.7. Dendrimer **13**

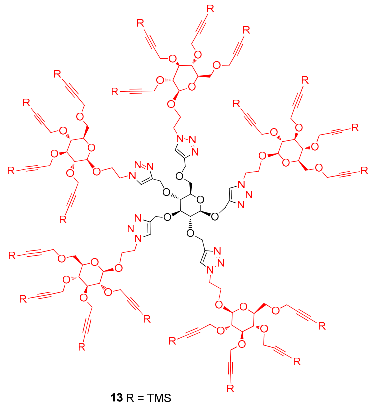

13 R = TMS

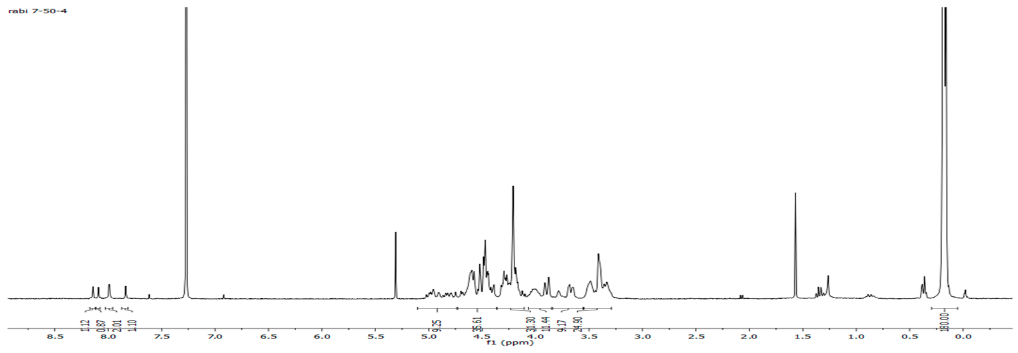

Figure S28. ^{1}H-NMR($CDCl_3$) of **13**.

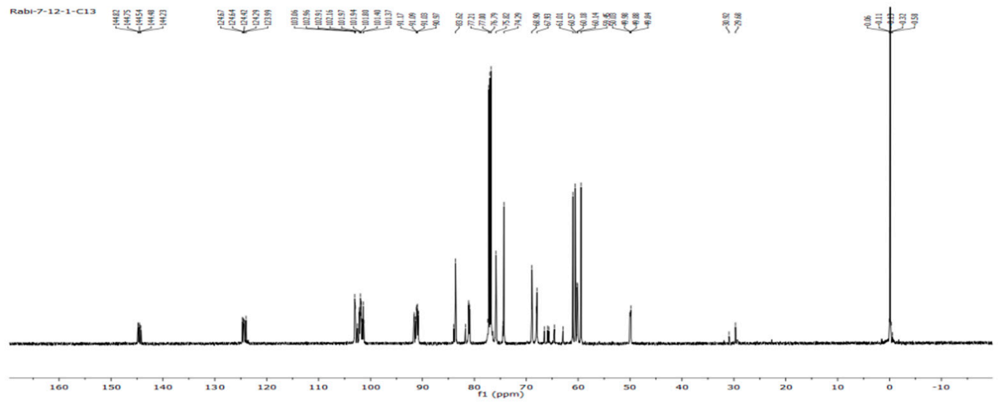

Figure S29. ^{13}C-NMR spectrum of **13**.

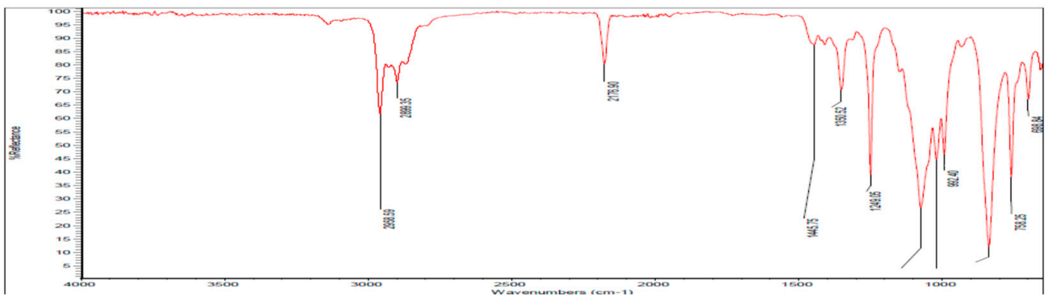

Figure S30. IR spectrum of **13**.

1.8. Dendrimer **14**

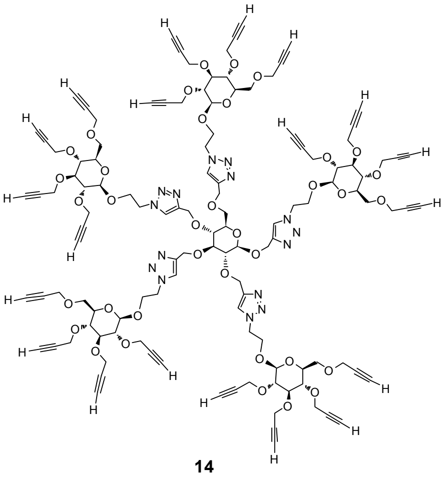

14

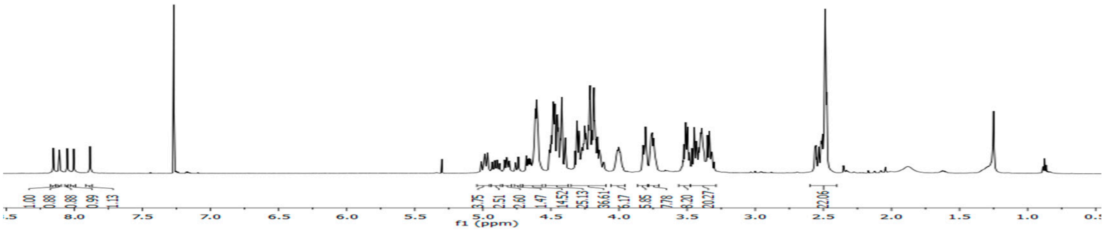

Figure S31. ¹H-NMR ($CDCl_3$) spectrum of **14**.

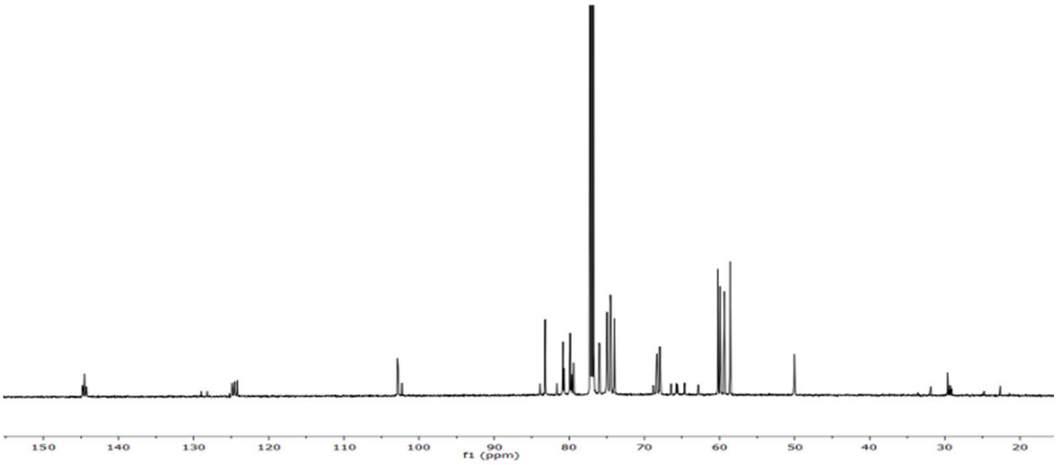

Figure S32. ¹³C-NMR spectrum of **14**.

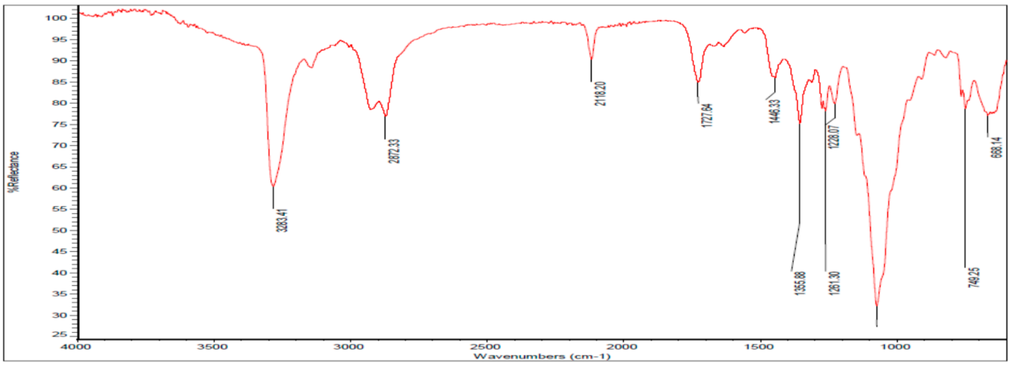

Figure S33. IR spectrum of **14**.

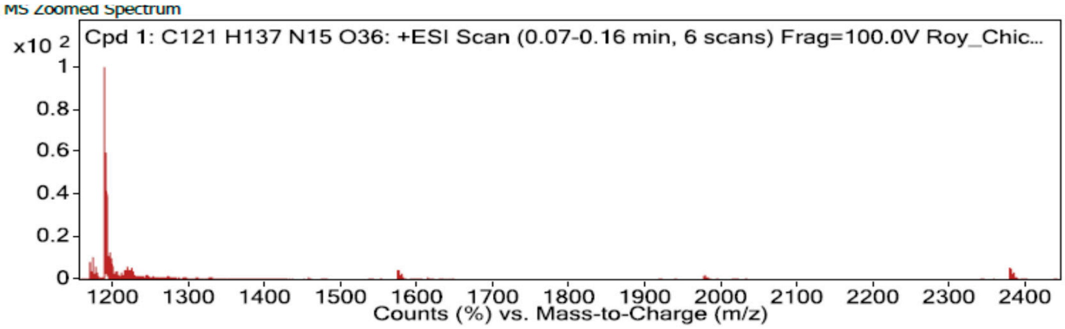

MS Spectrum Peak List

m/z	*Calc m/z*	Diff(ppm)	z	Abund	Formula	Ion
1185.9643				476		
1188.9781	1188.9748	2.76		27207	C121 H139 N15 O36	(M+2H)+2
1189.4779	1189.4764	1.3		41225	C121 H139 N15 O36	(M+2H)+2
1189.978	1189.9779	0.06		29687	C121 H139 N15 O36	(M+2H)+2
1190.4786	1190.4793	-0.58		16087	C121 H139 N15 O36	(M+2H)+2
1190.9772				22905		
1191.4753				25741		
1191.9767				17644		
1192.4771				9323		
1192.9761				7607		
1193.4741				7350		
1193.9739				4644		
1194.4713				2603		
2376.9488	2376.9615	-5.31	1	1715	C121 H139 N16 O35	(M+NH4)+[-H2O]
2377.9508	2377.9644	-5.74	1	2438	C121 H139 N16 O35	(M+NH4)+[-H2O]
2378.959	2378.9673	-3.52	1	1761	C121 H139 N16 O35	(M+NH4)+[-H2O]
2379.9581	2379.9702	-5.06	1	1032	C121 H139 N16 O35	(M+NH4)+[-H2O]
2393.9505	2393.9689	-7.69	1	289	C121 H141 N16 O36	(M+NH4)+
2398.9572	2398.9243	13.73	1	176	C121 H137 N15 Na O36	(M+Na)+
2414.9156	2414.8982	7.18	1	78	C121 H137 K N15 O36	(M+K)+

--- End Of Report ---

Figure S34. High resolution mass spectrum of **14**.

1.9. Dendrimer **15**

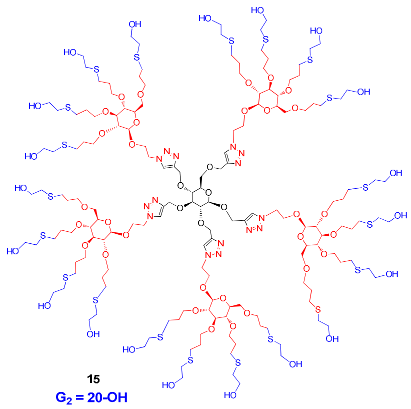

15

G_2 = 20-OH

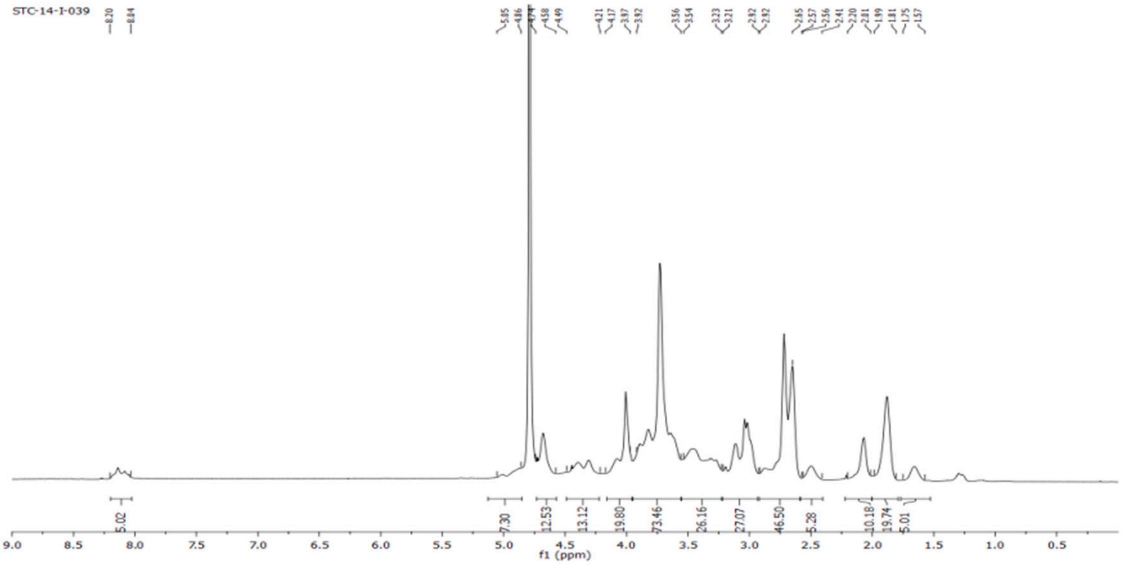

Figure S35. ^{1}H-NMR (D_2O) spectrum of **15**.

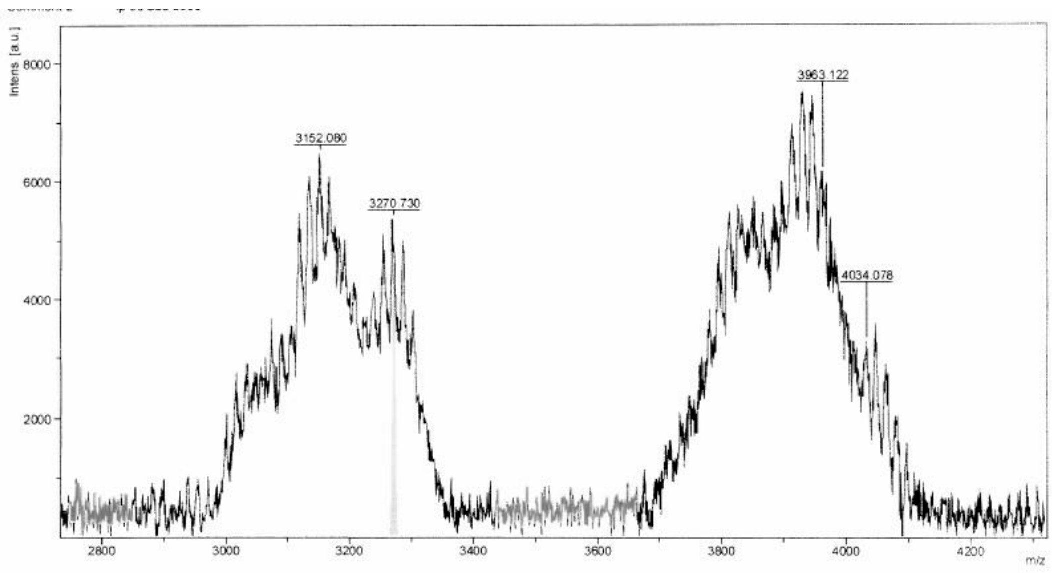

Figure S36. Maldi-tof of compound **15**.

1.10. Dendrimer **16**

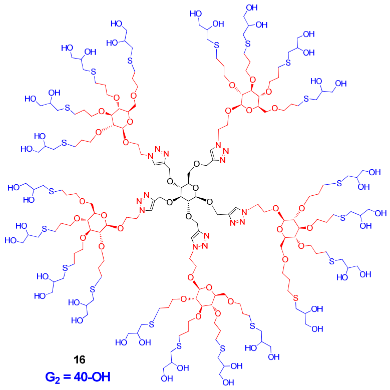

16
G_2 = 40-OH

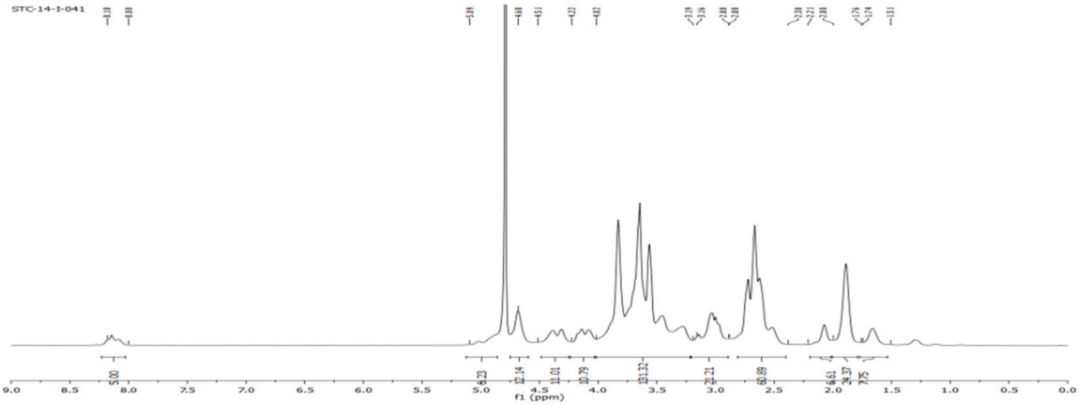

Figure S37. ^{1}H-NMR (D_2O) spectrum of **16**.

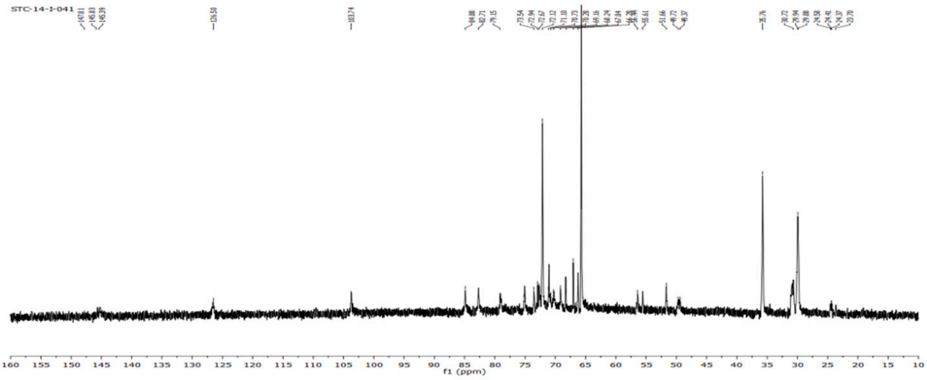

Figure S38. ^{13}C-NMR of **16**.

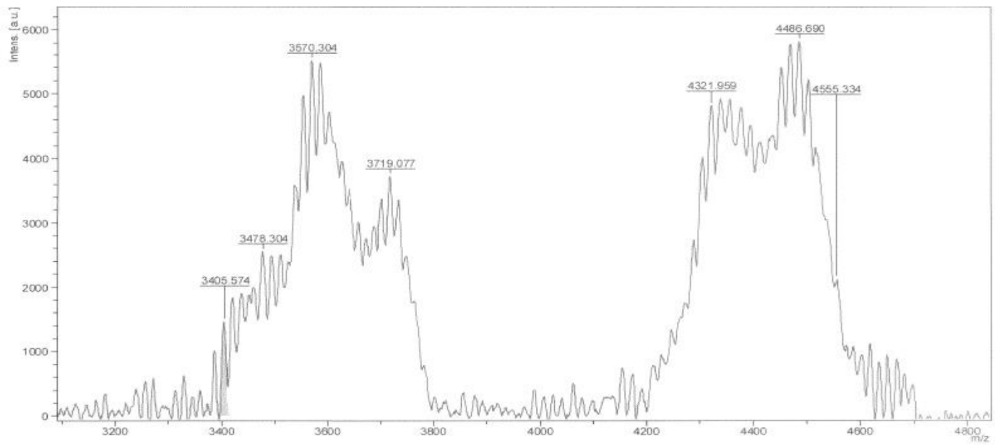

Figure S39. Maldi-tof of Compound **16**.

1.11. Dendrimer **17**

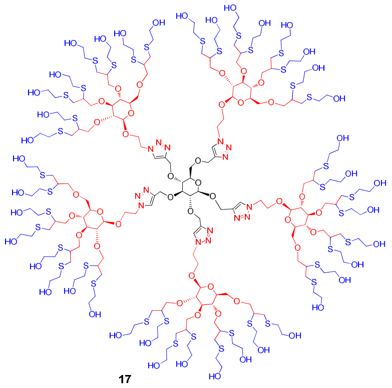

17

G_2 = 40-OH

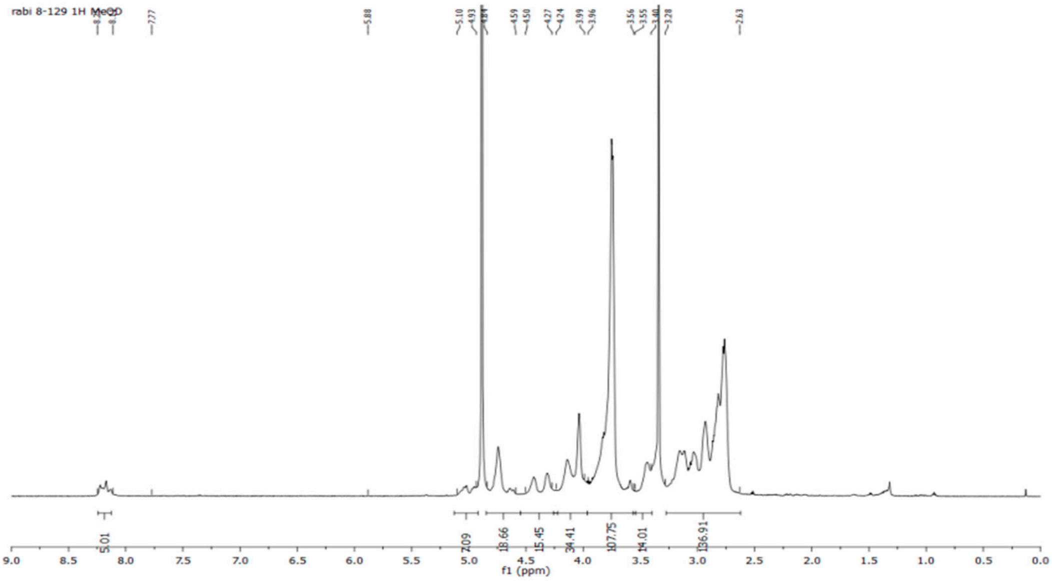

Figure S40. ^{1}H-NMR (D_2O) spectrum of **17**.

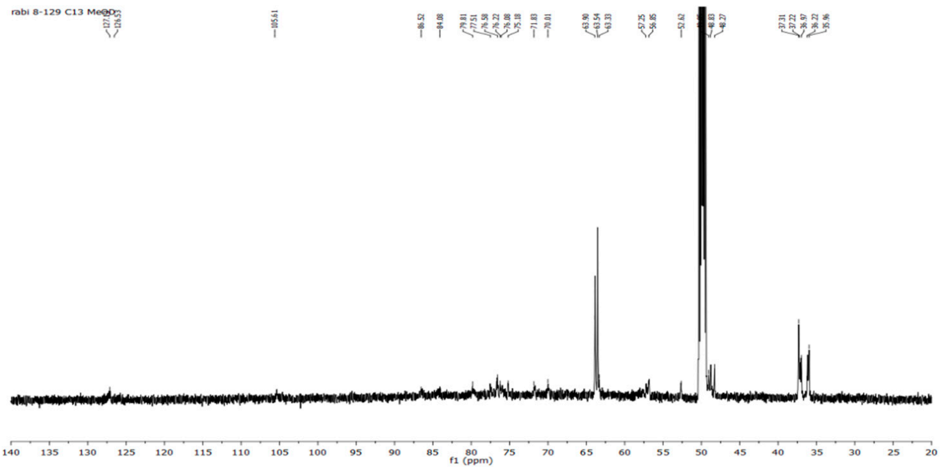

Figure S41. ^{13}C-NMR spectrum of **17**.

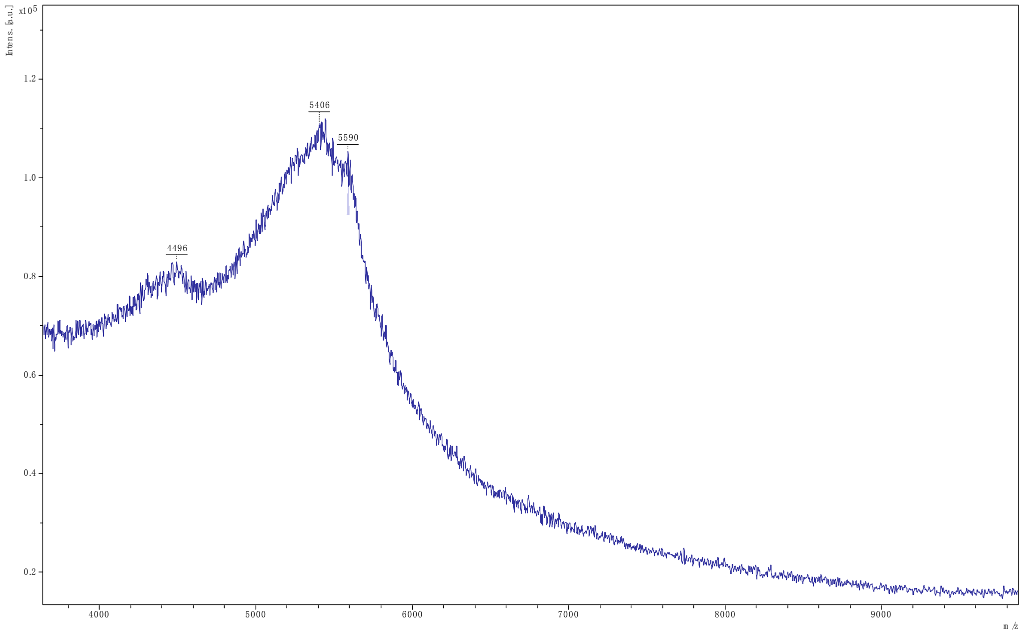

Figure S42. Maldi-tof of compound **17**.

1.12. Dendrimer **18**

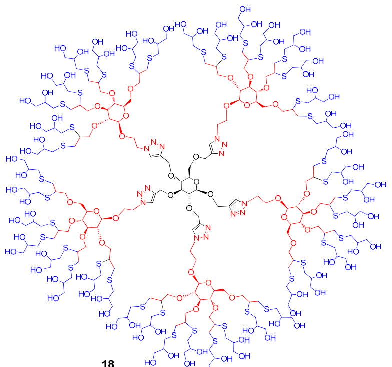

18

G_2 = 80-OH

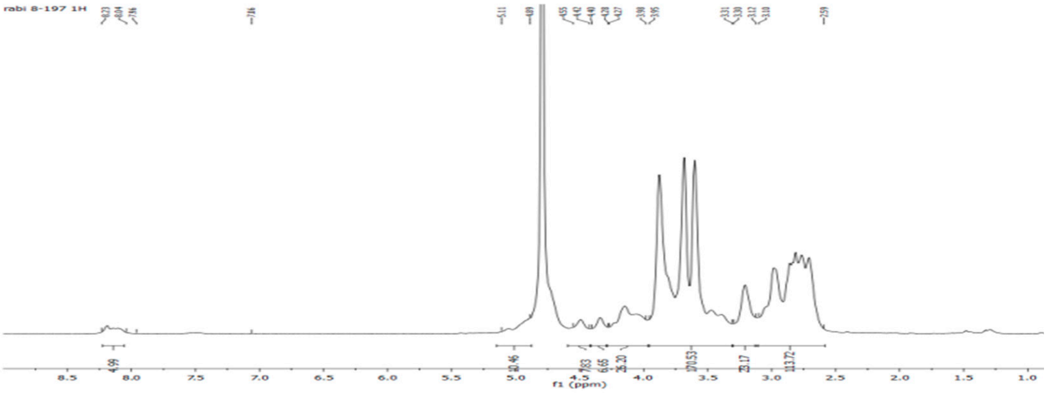

Figure S43. ^{1}H-NMR (D_2O) spectrum of **18**.

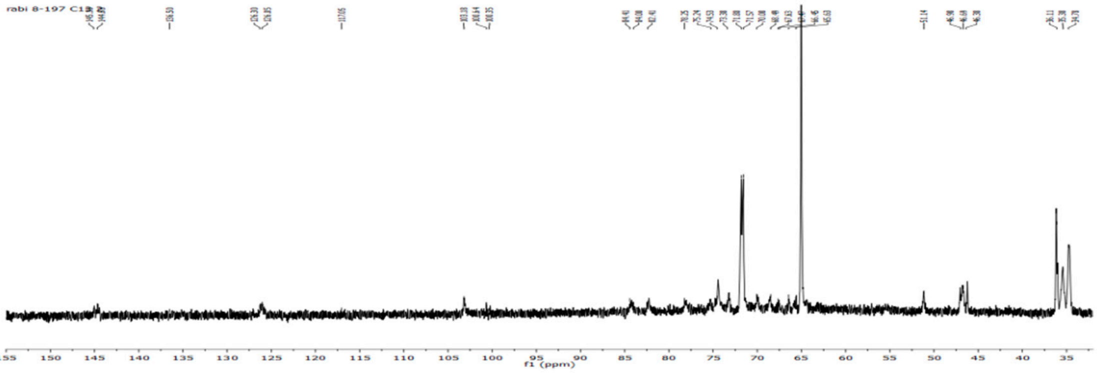

Figure S44. ^{13}C-NMR spectrum of **18**.

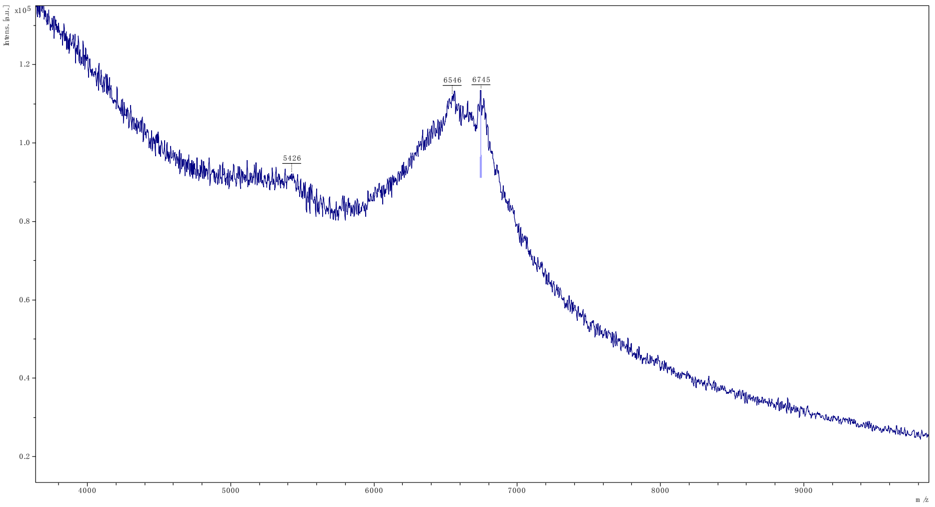

Figure S45. Maldi-tof of compound **18**.

1.13. Dendrimer **19**

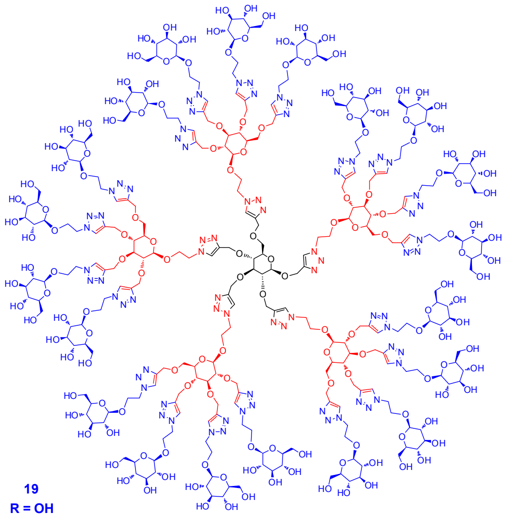

19
R = OH
G_2 = 80-OH

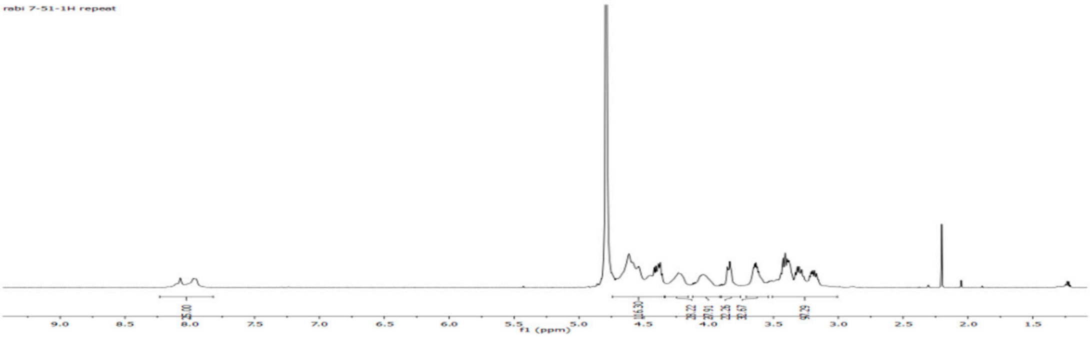

Figure S46. ^{1}H-NMR (D_2O) of **19**.

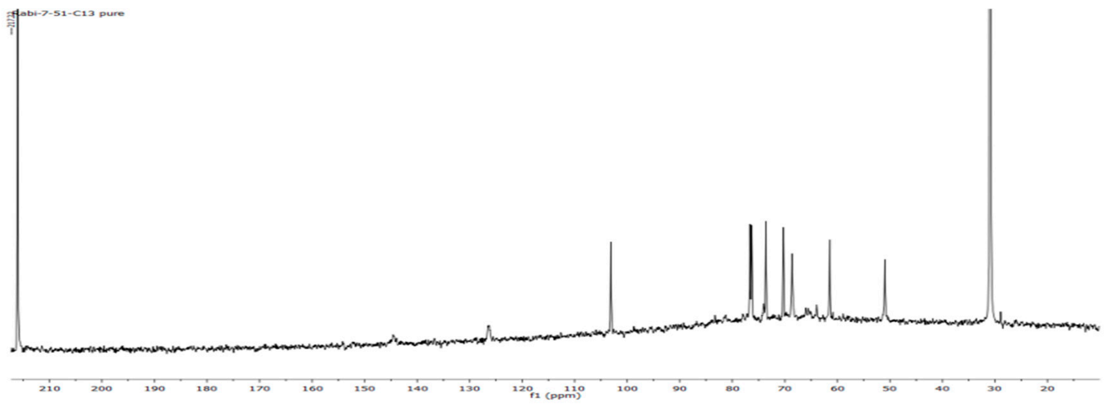

Figure S47. ^{13}C-NMR of **19**.

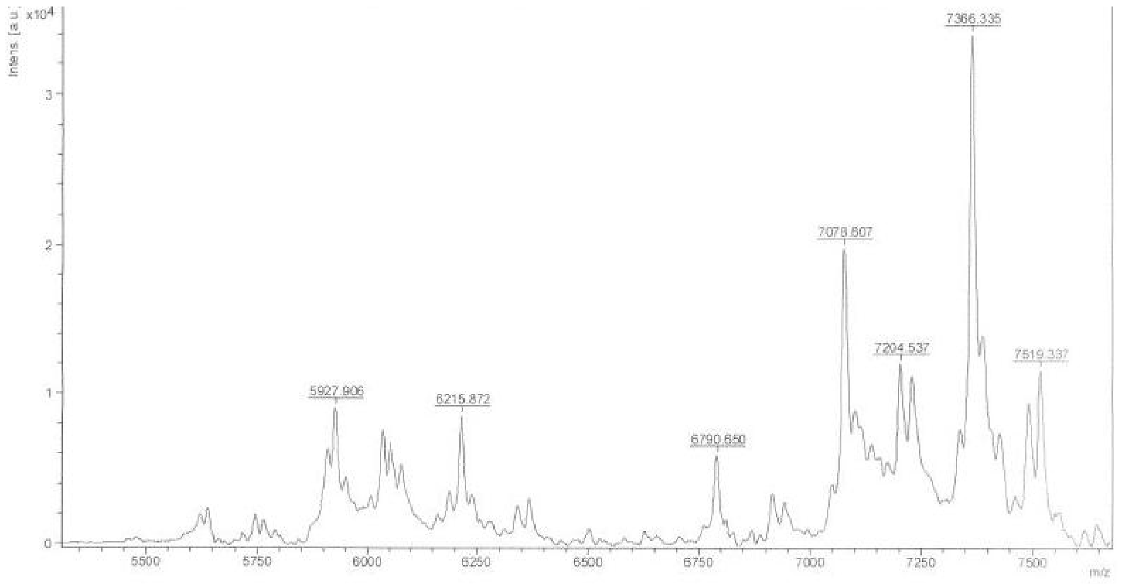

Figure S48. MALDI-TOF of Compound **19**.

1.14. Dendrimer **20**

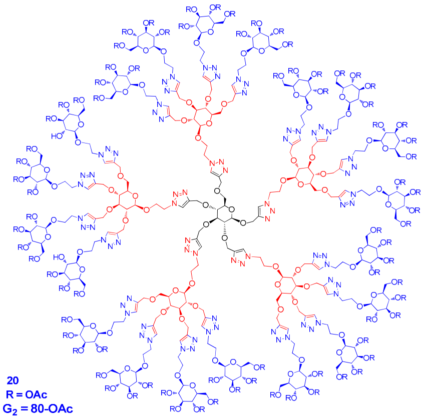

20
R = OAc
G_2 = 80-OAc

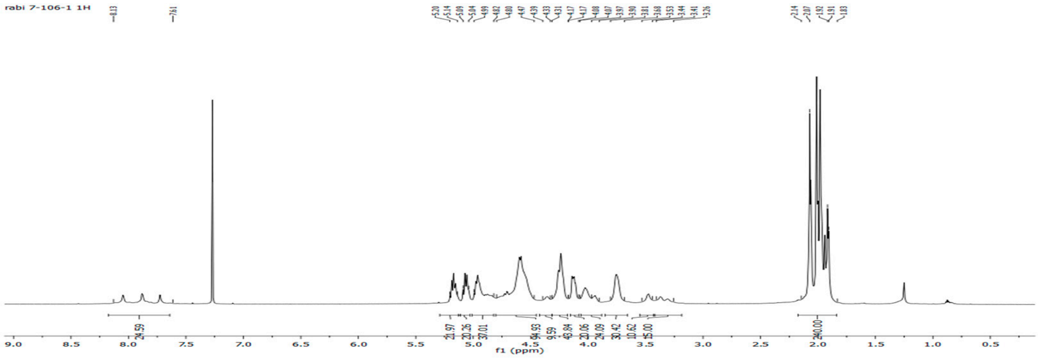

Figure S49. ^{1}H-NMR ($CDCl_3$) spectrum of **20**.

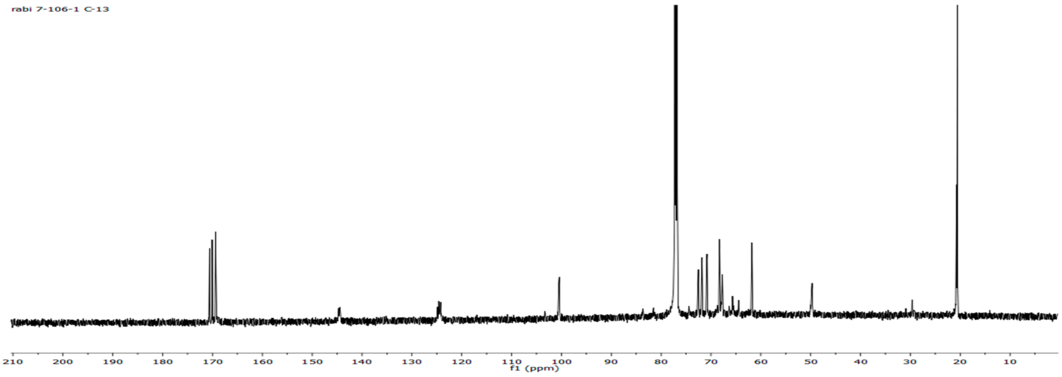

Figure S50. ^{13}C-NMR spectrum of **20**.

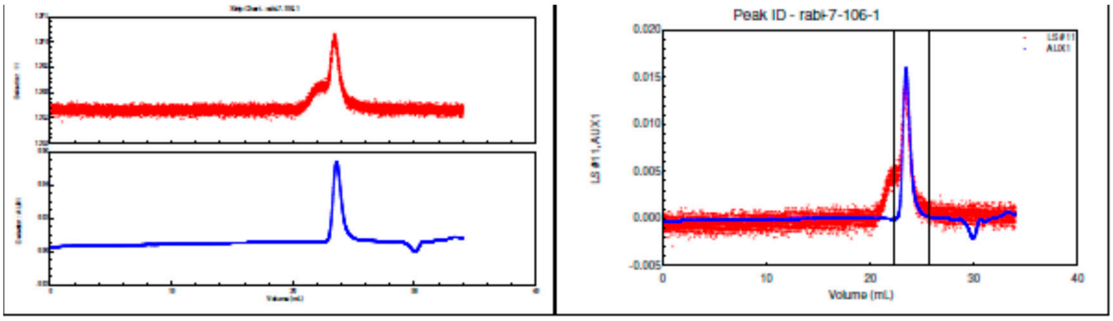

Figure S51. GPC of **20**.

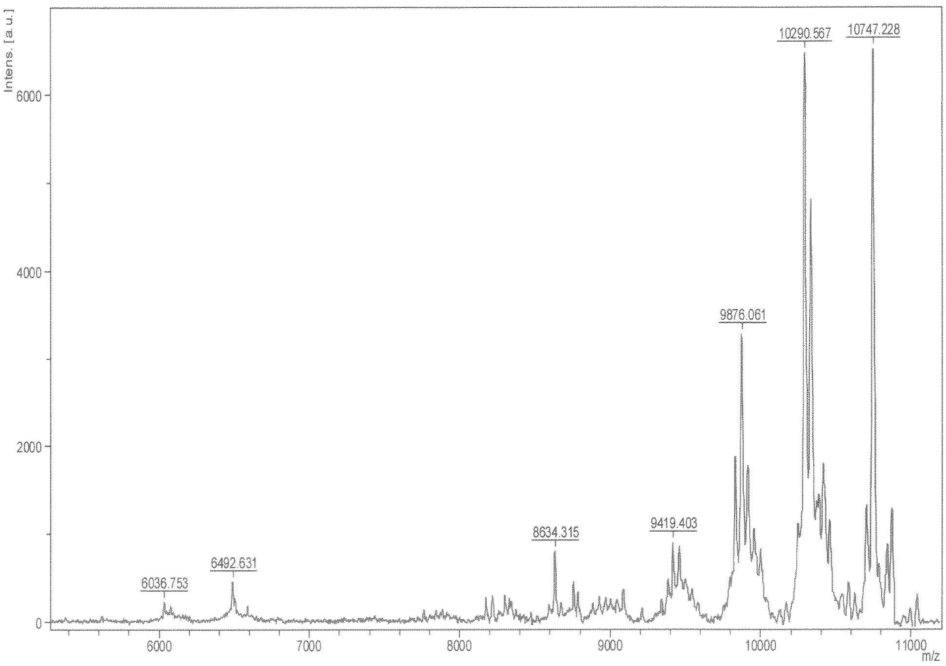

Figure S52. MALDI-TOF of Compound **20**.

1.15. Dendrimer **21**

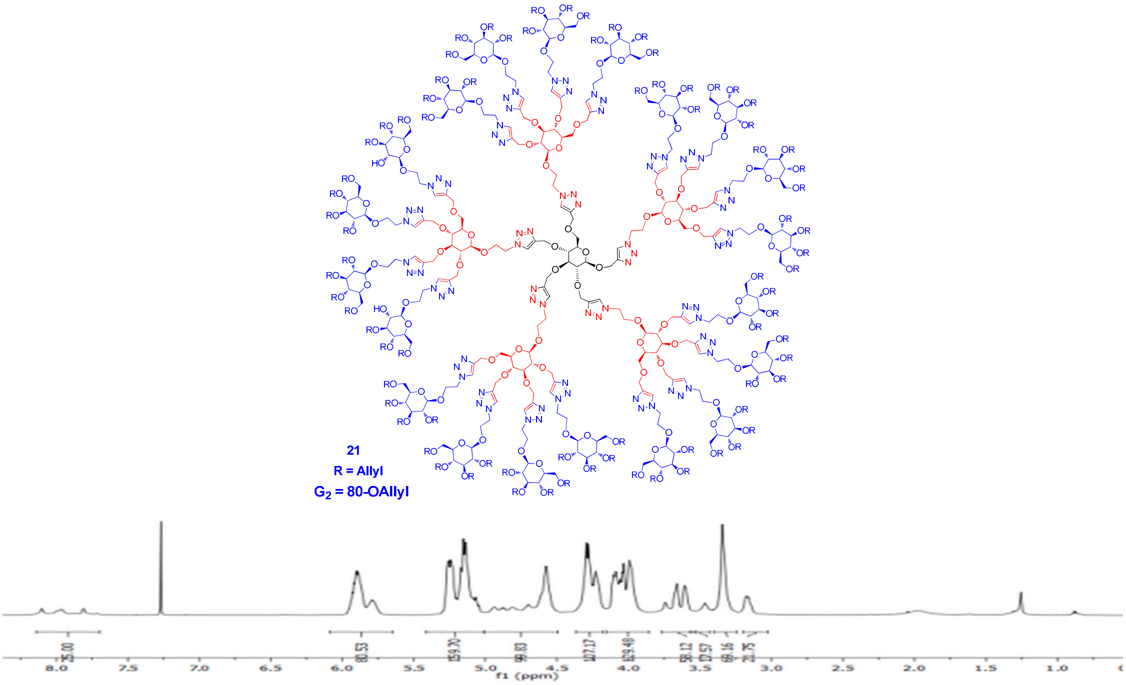

Figure S53. ^{1}H-NMR ($CDCl_3$) spectrum of **21**.

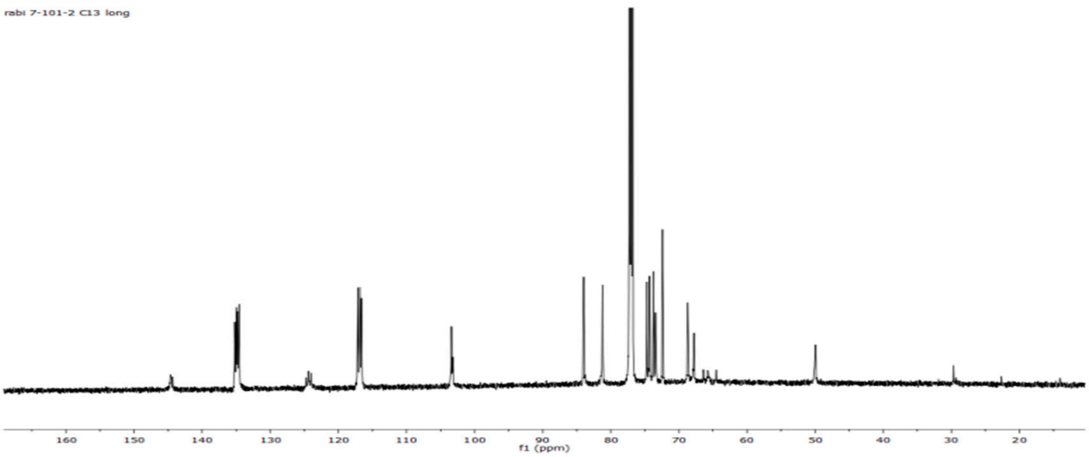

Figure S54. ^{13}C-NMR spectrum of **21**.

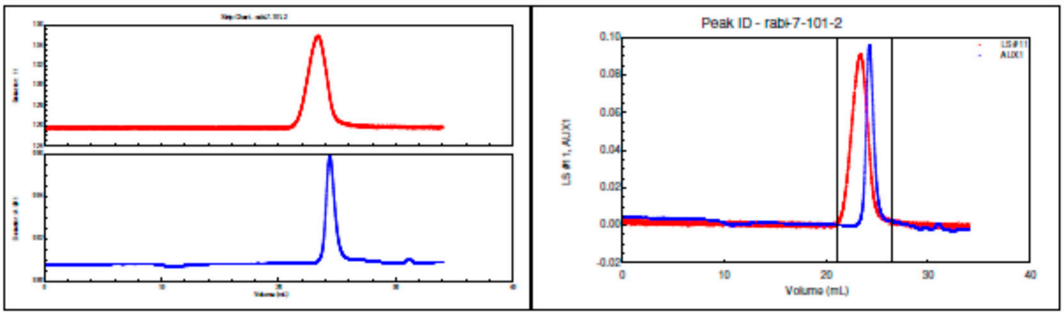

Figure S55. GPC of **21**.

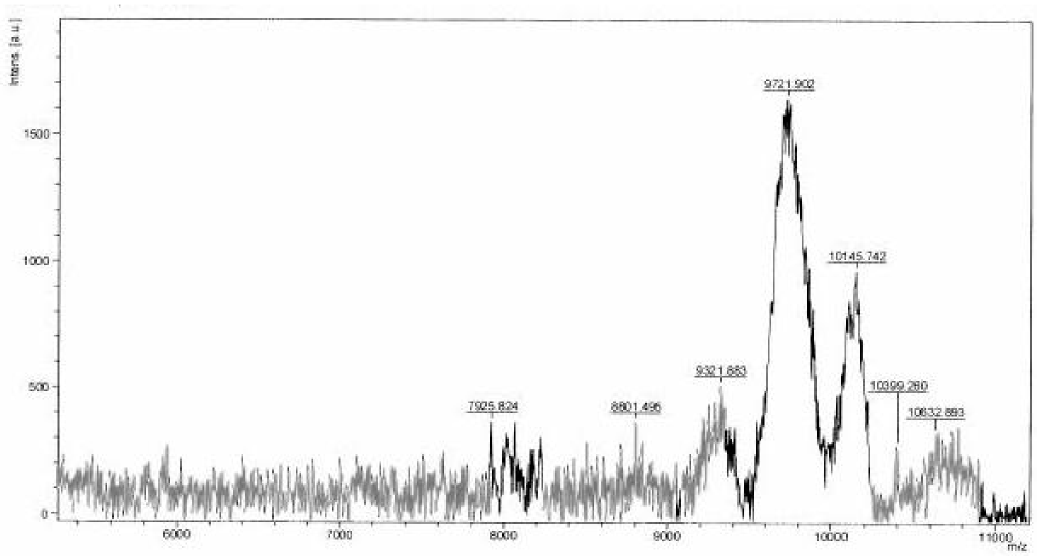

Figure S56. MALDI-TOF of Compound **21**.

2. Computational Methods

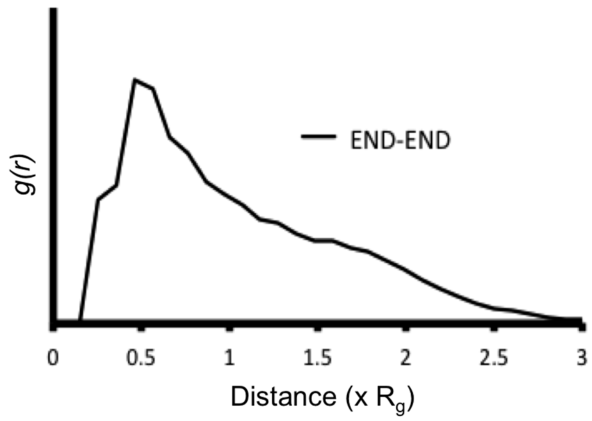

Figure S57. Radial distribution function (g(r)) of the END groups as a function of the distance. The most probable distance between surface groups in equilibrated **16** is at *c.a.* 0.5* R_g (i.e., ≈5 Å).

Table S1. Main structural features of dendrimer **16** in solution at the equilibrium.

R_g (Å)	END-END avg. Distance[a] (Å)	SASA (Å^2)	R_{SASA}[b] (Å)	V_{SASA}[c] (Å^3)	V_g[c] (Å^3)	V_{void}[d] (Å^3)	Porosity[e]
9.8	5	3332	16.3	18124	4017	14107	0.78

[a] Calculated from the position of the END-END *g(r)* peak (Figure S57). [b] Calculated from SASA, being SASA=4πR_{SASA}^2. [c] Calculated as the volume of the spheres having radius R_g ("full" volume) or R_{SASA} ("total" volume). [d] $V_{void} = V_{SASA} - V_g$. [e] Dendrimer's porosity is calculated as the ratio between void volume (V_{void}) over the total one (V_{SASA}).

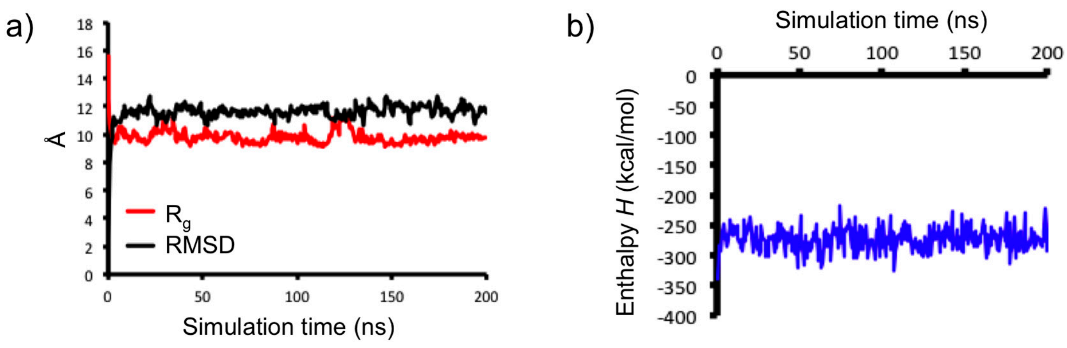

Figure S58. Equilibration of dendrimer **16** during the MD simulation. (**a**) Root mean square displacement (RMSD: black) and radius of gyration (R_g: red) data for **16** as a function of simulation time; (**b**) Enthalpy *H* of **16** calculated as the sum of solute-solute and solute-solvent interactions.
